# Selfee, self-supervised features extraction of animal behaviors

**DOI:** 10.7554/eLife.76218

**Published:** 2022-06-16

**Authors:** Yinjun Jia, Shuaishuai Li, Xuan Guo, Bo Lei, Junqiang Hu, Xiao-Hong Xu, Wei Zhang

**Affiliations:** 1 School of Life Sciences, IDG/McGovern Institute for Brain Research, Tsinghua University Beijing China; 2 Tsinghua-Peking Center for Life Sciences Beijing China; 3 Institute of Neuroscience, State Key Laboratory of Neuroscience, Chinese Academy of Sciences Center for Excellence in Brain Science and Intelligence Technology Shanghai China; 4 Shanghai Center for Brain Science and Brain-Inspired Intelligence Technology Shanghai China; MRC Laboratory of Molecular Biology United Kingdom; National Centre for Biological Sciences, Tata Institute of Fundamental Research India

**Keywords:** *Drosophila*, mouse, behavior, deep learning, *D. melanogaster*, Mouse

## Abstract

Fast and accurately characterizing animal behaviors is crucial for neuroscience research. Deep learning models are efficiently used in laboratories for behavior analysis. However, it has not been achieved to use an end-to-end unsupervised neural network to extract comprehensive and discriminative features directly from social behavior video frames for annotation and analysis purposes. Here, we report a self-supervised feature extraction (Selfee) convolutional neural network with multiple downstream applications to process video frames of animal behavior in an end-to-end way. Visualization and classification of the extracted features (Meta-representations) validate that Selfee processes animal behaviors in a way similar to human perception. We demonstrate that Meta-representations can be efficiently used to detect anomalous behaviors that are indiscernible to human observation and hint in-depth analysis. Furthermore, time-series analyses of Meta-representations reveal the temporal dynamics of animal behaviors. In conclusion, we present a self-supervised learning approach to extract comprehensive and discriminative features directly from raw video recordings of animal behaviors and demonstrate its potential usage for various downstream applications.

## Introduction

Extracting representative features of animal behaviors has long been an important strategy for studying the relationship between genes, neural circuits, and behaviors. Traditionally, human observations and descriptions are the primary solutions for animal behavior analysis (Hall, 1994; McGill, 1962; Rubenstein and Alcock, 2019). Well-trained researchers would define a set of behavior patterns and compare their intensity or proportion between experimental and control groups. With the emergence and flourish of machine learning methodology, supervised learning has been assisting human annotations and achieved impressive results (Jiang et al., 2019; Kabra et al., 2013; Segalin et al., 2020). Nevertheless, supervised learning is limited by prior knowledge (especially used for feature engineering) and manually assigned labels, thus could not identify behavioral features that are not annotated.

Other machine learning methods were then introduced to the field which was designed to extract representative features beyond human-defined labels. These methods can be generally divided into two major categories: one estimates animal postures with a group of pre-defined key points of the body parts, and the other directly transforms raw images. The former category marks representative key points of animal bodies, including limbs, joints, trunks, and/or other body parts of interest (Graving et al., 2019; Günel et al., 2019; Mathis et al., 2018). Those features are usually sufficient to represent animal behaviors. However, it has been demonstrated that the key points generated by pose estimation are less effective for direct behavior classification or two-dimensional visualization (Luxem et al., 2020; Sun et al., 2021). Sophisticated post-processing like recurrent neural networks (RNNs) (Luxem et al., 2020), non-locomotor movement decomposition (Huang et al., 2021), or feature engineering (Sun et al., 2021) can be applied to transform the key points into higher-level discriminative features. Additionally, the neglection of body parts could cause problems. For example, the position of the proboscis of a fly is commonly neglected in behavior studies using pose estimation software (Calhoun et al., 2019; Sun et al., 2021). Still, it is crucial for feeding (Zhou et al., 2019), licking behavior during courtship (Mezzera et al., 2020), and hardness detection for a substrate (Zhang et al., 2020). Finally, best to our knowledge, there is no demonstration of these pose-estimation methods applied to multiple animals of the same color with intensive interactions. Thus, the application of pose estimation to mating behaviors of two black mice, a broadly adopted behavior paradigm (Bayless et al., 2019; Wei et al., 2018; Zhang et al., 2021), could be limited because labeling body parts during mice mounting is challenging even for humans (see Discussion for more details). Therefore, using these feature extraction methods requires rigorously controlled experimental settings, additional feature engineering, and considerable prior knowledge of particular behaviors.

In contrast, the other category transforms pixel-level information without key point labeling, thus retaining more details and requiring less prior knowledge. Feature extraction of images could be achieved by wavelet transforms (Wiltschko et al., 2015) or Radon transforms Berman et al., 2014; Ravbar et al., 2019 followed by principal component analysis (PCA), and these transforms can be applied to either 2D images or depth images. However, preprocessing such as segmentation and/or registration of the images is required to achieve spatial invariance in some implementations, a task that is particularly difficult for multi-agent videos (one method named ABRS solved this problem by using the spectra of Radon transforms, which is translation and rotation invariant; Ravbar et al., 2019). Beyond translation or rotation invariance, the relative position between animals is also important to social behaviors. Animals could be of varies of relative positions when perform the same type of behaviors. Extracted features should be invariant to these variations and capture the major characteristics. Additionally, because these methods usually use unlearnable transforms, although it makes them highly transferrable from dataset to dataset, they could not be adaptive to images with different characteristics and thus select the most discriminative and relevant features across the dataset automatically. Flourished deep learning methods, especially convolutional neural networks (CNNs) (Lecun et al., 1998), could adaptively extract features from diversified datasets. Also, they have been proven more potent than classic computer vision algorithms like wavelet transforms (Romero et al., 2009) and Radon transforms (Aradhya et al., 2007) on a famous grayscale dataset MNIST, even without supervising (Ji et al., 2019). Therefore, we attempt to adopt CNNs to achieve end-to-end feature extractions of animal behaviors that are comprehensive and discriminative.

The cutting-edge self-supervised deep learning methods aim to extract representative features for downstream missions by comparing different augmentations of the same image and/or different images (Caron et al., 2020; Chen et al., 2020; Grill et al., 2020; He et al., 2020; Wu et al., 2018). Compared with previous techniques, these methods have three major advantages. First, self-supervised or unsupervised methods could completely avoid human biases. Second, the augmentations used to create positive samples promise invariance of the neural networks to object sizes, spatial orientations, and ambient laminations so that registration or other preprocessing is not required. Finally, the networks are optimized to export similar results for positive samples and separate negative ones, such that the extracted features are inherently discriminative. Even without negative samples, the networks can utilize differential information within batches to obtain remarkable results on downstream missions like classification or image segmentation (Chen and He, 2021; Grill et al., 2020; Zbontar et al., 2021). These advances in self-supervised learning provide a promising way to analyze animal behaviors.

In this work, we develop Selfee (**Sel**f-supervised **Fe**atures **E**xtraction) that adopts recently published self-supervised learning algorithms and CNNs to analyze animal behaviors. Selfee is trained on massive unlabeled behavior video frames (around five million frames from hundreds of videos) to avoid human bias in annotating animal behaviors, and it could capture a global character of animal behaviors even when detailed postures are hard to extract, similar to human perception. During the training process, Selfee learns to project images to a low-dimensional space without being affected by shooting conditions, image translation, and rotation, where cosine distance is proper to measure the similarities of original pictures. Selfee also provides potential for various downstream analyses. We demonstrate that the extracted features are suitable for t-SNE visualization, *k*-NN-based classification, *k*-NN-based anomaly detection, and dynamic time warping (DTW). We also show that further integrated modeling, like the autoregressive hidden Markov model (AR-HMM), is compatible with Selfee extracted Meta-representations. We apply Selfee to fruit flies, mice, and rats, three widely used model animals, and validate our results with manual annotations or pre-existed animal tracking methods. Discoveries of behavioral phenotypes in mutant flies by Selfee are consistent with either human observations or other animal tracking analysis, and can be validated by biological experiments. The performance of Selfee on these model species indicates its potential usage for behavioral studies of non-model animals as well as other tasks. We also provide an open-source Python project and pre-trained models of flies and mice to the community (see more in Code Availability).

## Results

### Workflow of Selfee and its downstream analyses

**Figure 1.**
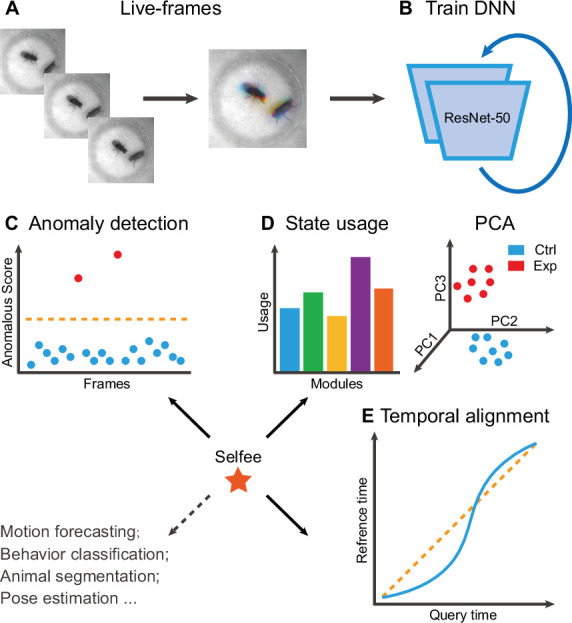
The framework of Selfee (**Sel**f-supervised **Fe**atures **E**xtraction) and its downstream applications. (**A**) One live-frame is composed of three tandem frames in R, G, and B channels, respectively. The live-frame could capture the dynamics of animal behaviors. (**B**) Live-frames are used to train Selfee, which adopts a backbone of ResNet-50. (C, D, and E) Representations produced by Selfee could be used for anomaly detection that could identify unusual animal postures in the query video compared with the reference videos. (**C**) AR-HMM (autoregressive hidden Markov model) that models the local temporal characteristics of behaviors and clusters frames into modules (states) and calculates stages usages of different genotypes (**D**) DTW (dynamic time warping) that aligns behavior videos to reveal differences of long-term dynamics (**E**) and other potential tasks including behavior classification, forecasting, or even image segmentation and pose estimation after appropriately modifying and fine-tuning of the neural networks.

Selfee is trained to generate Meta-representations at the frame level, then analyzed at different time scales. First, grayscale videos are decomposed into single frames, and three tandem frames are stacked into a live-frame to generate a motion-colored RGB picture (Figure 1A). These live-frames preserve not only spatial information (e.g., postures of each individual or relative distances and angles between individuals) within each channel but also temporal information across different channels. Live-frames are used to train Selfee to produce comprehensive and discriminative representations at the frame level (Figure 1B). These representations can be later used in numerous applications. For example, anomaly detection on mutant animals can discover new phenotypes compared with their genetic controls (Figure 1C). Also, the AR-HMM could be applied to model the micro-dynamics of behaviors, such as the duration of states or the probabilities of state transitions (Wiltschko et al., 2015). The AR-HMM splits videos into modules and yields behavioral state usages that visualize differences between genotypes (Figure 1D). In contrast, DTW could compare the long-term dynamics of animal behaviors and capture global differences at the video level (Myers et al., 1980) by aligning pairs of time series and calculating their similarities (Figure 1E). These three demonstrations cover different time scales from frame to video level, and other downstream analyses could also be incorporated into the workflow of Selfee.

**Figure 1—figure supplement 1.**
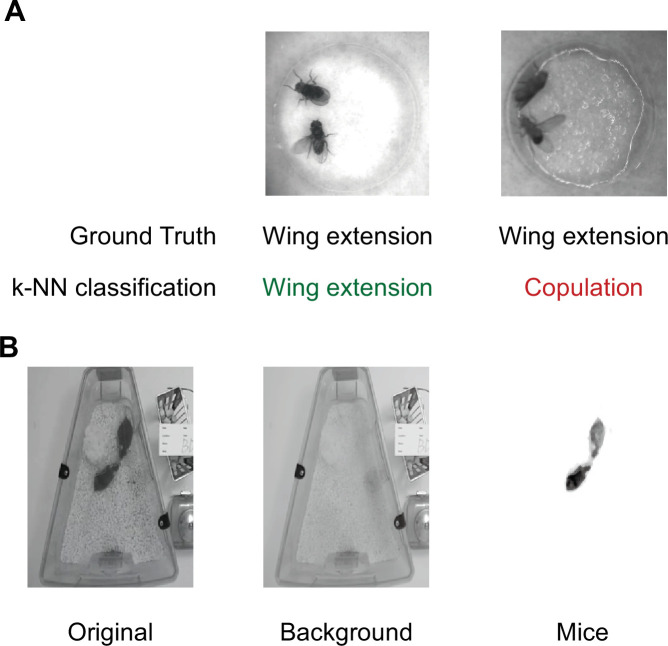
Beddings and backgrounds that affect training and inference of Selfee (**Sel**f-supervised **Fe**atures **E**xtraction). (**A**) Textures on the damped filter paper would mislead Selfee to output features similar to copulation but not wing extension (ground truth). The left example showed a background that would not affect Selfee neural network, and the right example showed a background that could strongly affect classification accuracy. (**B**) Background inconsistency would affect the training process when Selfee was applied to mice behavior data. Therefore, backgrounds were removed from all frames to avoid potential defects. After background removal, illumination normalization was applied.

**Figure 1—figure supplement 2.**
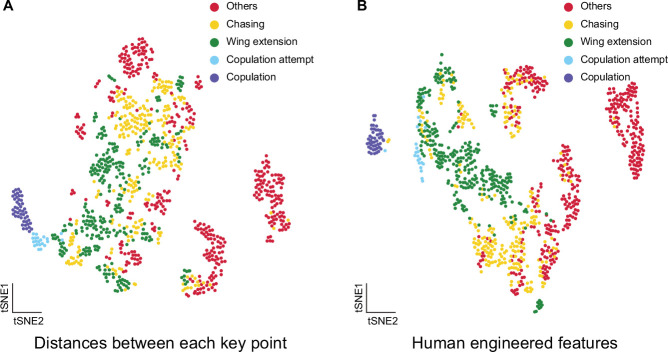
t-SNE visualization of pose estimation derived features. (**A**) Visualization of fly courtship live-frames with t-SNE dimension reduction of distances between key points, including head, tail, thorax, and wings. Each dot was colored based on human annotations. Points representing non-interactive behaviors (‘others’), chasing, wing extension, copulation attempt, and copulation were colored with red, yellow, green, blue, and violet, respectively. (**B**) Visualization of fly courtship live-frames with t-SNE dimension reduction of human-engineered features, including male head to female tail distance, male body to female body distance, male wing angle, female wing angle, and angle between male body axis. Each dot was colored based on human annotations. Points representing non-interactive behaviors (‘others’), chasing, wing extension, copulation attempt, and copulation were colored with red, yellow, green, blue, and violet, respectively.

**Figure 1—figure supplement 3.**
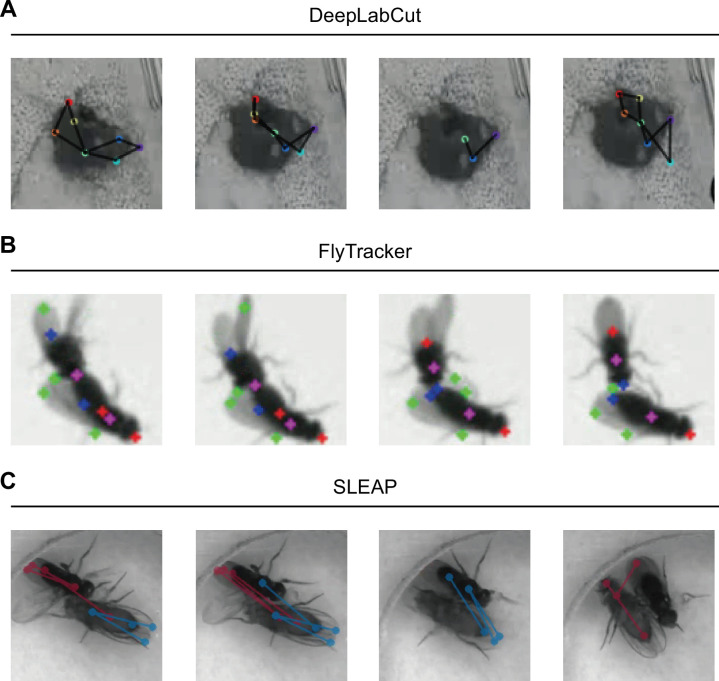
Animal tracking with DLC, FlyTracker, and SLEAP. (**A**) Visualization of DLC tracking results on intensive interactions between mice during mating behavior. The nose, ears, body center, hips, and bottom were labeled. DLC tended to detect two animals as one due to occluding. (**B**) Visualization of FlyTracker tracking results provided in Fly-vs-Fly dataset. The head, tail, center, and wings were colored in red, blue, purple, and green, respectively. When two flies were close, wings became hard to be detected correctly. (**C**) Visualization of SLEAP tracking results on close interactions during fly courtship behavior. Five body parts were marked with points, including head, tail, thorax, and wings, and head to tail, thorax to wings were linked by lines. Female was labeled in red, and the male was in blue. Body parts were wrongly assigned when two animals were close.

**Figure 1—video 1.** Visualization of DLC tracking results on intensive interactions between mice during mating behavior. The nose, ears, body center, hips, and bottom were labeled. DLC worked great when two animals were separated, but it tended to detect two animals as one during mounting or intromission due to occluding.

**Figure 1—video 2.** A tracking example of FlyTracker of Fly-vs-Fly dataset. Visualization of FlyTracker tracking results provided in Fly-vs-Fly dataset. The head, tail, center, and wings were colored in red, blue, purple, and green, respectively. The tracking result was very competent even compared with deep learning-based methods. However, when two flies were close, wings, even bodies, became hard to be detected correctly.

**Figure 1—video 3.** A tracking example of SLEAP on fly courtship behavior. Visualization of SLEAP tracking results on fly courtship behavior. Five body parts were marked with points, including head, tail, thorax, and wings, and head to tail, thorax to wings were linked by lines. Female was labeled in red, and the male was in blue. In general, the tracking result was good. However, body parts were wrongly assigned when two animals were close, and performance was significantly impaired during copulation attempt and copulation.

Compared with previous machine learning frameworks for animal behavior analysis, Selfee has three major advantages. First, Selfee and the Meta-representations could be used for various tasks. The contrastive learning process of Selfee would allow output features to be appropriately compared by cosine similarity. Therefore, distance-based applications, including classification, clustering, and anomaly detection, would be easily realized. It was also reported that with some adjustment of backbones, self-supervised learning would facilitate tasks such as pose estimation (Dahiya et al., 2021) and object segmentation (Caron et al., 2021; He et al., 2020). Those findings indicate that Selfee could be generalized, modified, and fine-tuned for animal pose estimation or segmentation tasks. Second, Selfee is a fully unsupervised method developed to annotate animal behaviors. Although some other techniques also adopt semi-supervised or unsupervised learning, they usually require manually labeled pre-defined key points of the images (Huang et al., 2021; Luxem et al., 2020); some methods also require expert-defined programs for better performance (Sun et al., 2021). Key point selection and program incorporation require a significant amount of prior knowledge and are subject to human bias. In contrast, Selfee does not need any prior knowledge. Finally, Selfee is relatively hardware-inexpensive compared with other self-supervised learning methods (Chen et al., 2020; Chen et al., 2020; Grill et al., 2020). Training Selfee only takes 8 hr on a single RTX 3090 GPU (graphic card), and the inference speed could reach 800 frames per second. Selfee could accept top-view 2D grayscale video frames as inputs so that neither depth cameras (Wiltschko et al., 2015) nor fine-calibrated multi-view camera arrays (Huang et al., 2021) are required. Therefore, Selfee can be trained and used with routinely collected behavior videos on ordinary desktop workstations, warranting its accessibility to biology laboratories.

### Siamese CNNs capture discriminative representations of animal posture

**Figure 2.**
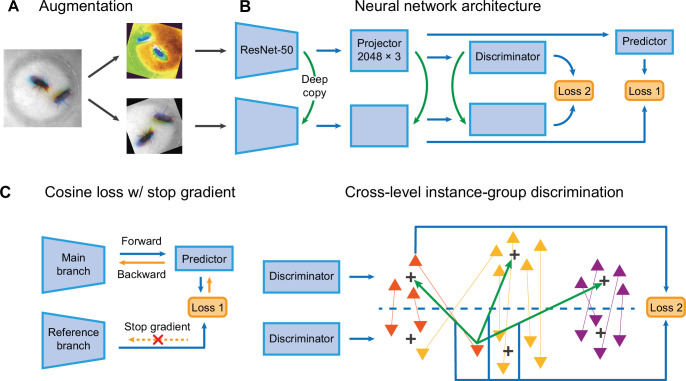
The network structure of Selfee (**Sel**f-supervised **Fe**atures **E**xtraction). (**A**) The architecture of Selfee networks. Each live-frame is randomly transformed twice before being fed into Selfee. Data augmentations include crop, rotation, flip, Turbo, and color jitter. (**B**) Selfee adopts a SimSiam-style network structure with additional group discriminators. Loss 1 is canonical negative cosine loss, and loss 2 is the newly proposed CLD (cross-level instance-group discrimination) loss. (**C**) A brief illustration of two loss terms used in Selfee. The first term of loss is negative cosine loss, and the outcome from the reference branch is detached from the computational graph to prevent mode collapse. The second term of loss is the CLD loss. All data points are colored based on the clustering result of the upper branch, and points representing the same instance are attached by lines. For one instance, the orange triangle, its representation from one branch is compared with cluster centroids of another branch and yields affinities (green arrows). Loss 2 is calculated as the cross-entropy between the affinity vector and the cluster label of its counterpart (blue arrows).

Selfee contains a pair of Siamese CNNs trained to generate discriminative representations for live-frames. ResNet-50 (He et al., 2016) is chosen as the backbone whose classifier layer is replaced by a three-layer multi-layer perceptron (MLP). These MLPs are called projectors which yield final representations during the inference stage. There are two branches in Selfee. The main branch is equipped with an additional predictor, while the reference branch is a copy of the main branch (the SimSiam style; Chen et al., 2020). To the best of our knowledge, the SimSiam style is the most straightforward Siamese CNN frameworks with only one term of loss. Both branches contain group discriminators after projectors and perform dimension reduction on extracted features for online clustering (Figure 2B).

**Figure 2—figure supplement 1.**
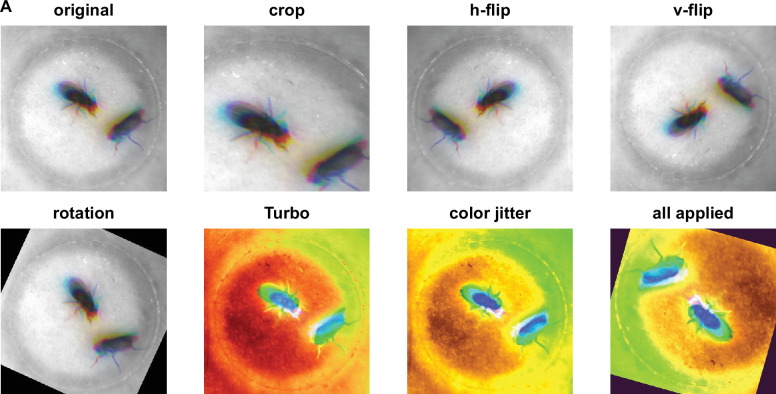
Different augmentations used for Selfee (**Sel**f-supervised **Fe**atures **E**xtraction) training. (**A**) Visualization of each augmentation. For data augmentations, crop, rotation, flip, Turbo, and color jitter were applied. Each live-frame was randomly cropped into a smaller version containing more than 49% (70%×70%) of the original image; then the image was randomly (clockwise or anticlockwise) rotated for an angle smaller than the acute angle formed by the diagonal line and the vertical line, then the image would be vertically flipped, horizontally flipped, and/or applied the Turbo lookup table at the probability of 50%, respectively; and finally, the brightness, contrast, saturation, and hue were randomly adjusted within 10% variation. Detailed descriptions of each augmentation could be found in the Materials and methods and source codes.

During the training stage, batches of live-frames are randomly transformed twice and fed into the main branch and reference branch, respectively. Augmentations applied to live-frames include crop, rotation, flip, and application of the Turbo lookup table (Mikhailov, 2019) followed by color jitters (Figure 2A, Figure 2—figure supplement 1). The reference branch yields a representation of received frames, while the main branch predicts the outcome of the reference branch. This is the first objective of the training process, which optimizes the cosine similarity between the outcome of the reference branch and its prediction given by the main branch. To prevent mode collapse, the reference branch will not receive gradient information during optimization, which means the outcome of the reference branch is detached from the computational graph and treated as ground truth (Figure 2C). The second objective is to optimize the clustering results of representations from two discriminators. For one instance, its representation from one branch is compared with cluster centroids of another branch and yields affinities. The affinity vector is optimized to be consistent with the cluster label of its counterpart. In the original publication (Wang et al., 2021), this loss is termed cross-level instance-group discrimination (CLD) loss (Figure 2C). Because the main branch and reference branch are symmetric, similar loss calculations can also be done after swapping the identity of these two branches. The final loss is the average loss under the two conditions. In this way, Selfee is trained to be invariant to those transforms and focus on critical information to yield discriminative representations.

**Figure 3.**
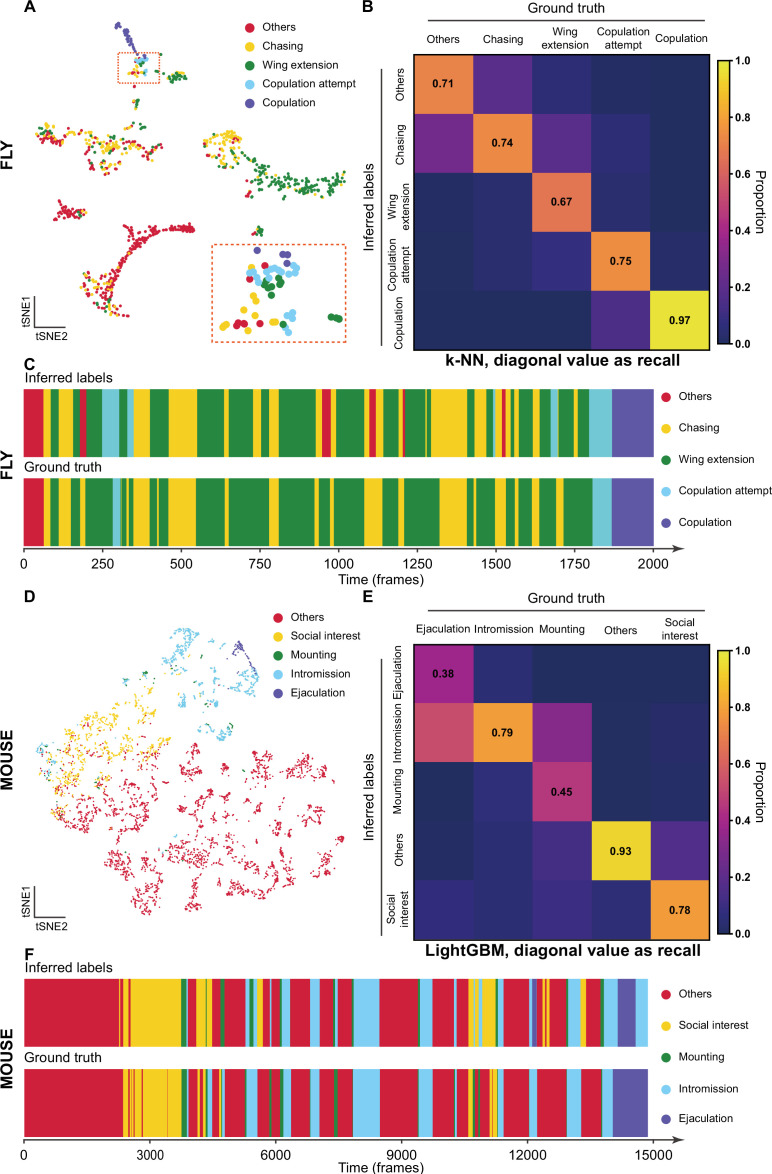
The validation of Selfee (**Sel**f-supervised **Fe**atures **E**xtraction) with human annotations. (**A**) Visualization of fly courtship live-frames with t-SNE dimension reduction. Each dot was colored based on human annotations. Points representing chasing, wing extension, copulation attempt, copulation, and non-interactive behaviors (‘others’) were colored with yellow, green, blue, violet and red, respectively. (**B**) The confusion matrix of the *k*-NN classifier for fly courtship behavior, normalized by the numbers of each behavior in the ground truth. The average *F*_1_ score of the sevenfold cross-validation was 72.4%, and mAP was 75.8%. The recall of each class of behaviors was indicated on the diagonal of the confusion matrix. (**C**) A visualized comparison of labels produced by the *k*-NN classifier and human annotations of fly courtship behaviors. The *k*-NN classifier was constructed with data and labels of all seven videos used in the cross-validation, and the *F*_1_ score was 76.1% and mAP was 76.1%. (**D**) Visualization of live-frames of mice mating behaviors with t-SNE dimension reduction. Each dot is colored based on human annotations. Points representing non-interactive behaviors (‘others’), social interest, mounting, intromission, and ejaculation were colored with red, yellow, green, blue, and violet, respectively. (**E**) The confusion matrix of the LightGBM (Light Gradient Boosting Machine) classifier for mice mating behaviors, normalized by the numbers of each behavior in the ground truth. For the LightGBM classifier, the average *F*_1_ score of the eightfold cross-validation was 67.4%, and mAP was 69.1%. The recall of each class of behaviors was indicated on the diagonal of the confusion matrix. (**F**) A visualized comparison of labels produced by the LightGBM classifier and human annotations of mice mating behaviors. An ensemble of eight trained LightGBM was used, and the *F*_1_ sore was 68.1% and mAP was not available for this ensembled classifier due to the voting mechanism.

After the training stage, we evaluated the performance of Selfee with t-SNE visualization and *k*-NN classification. To investigate whether our model captured human-interpretable features, we manually labeled one clip of *Drosophila* courtship video and visualized those representations with t-SNE dimension reduction. On the t-SNE map, human-annotated courtship behaviors, including chasing, wing extension, copulation attempt, copulation, and non-interactive behaviors (‘others’), were grouped in a non-random pattern (Figure 3A). Then, we would like to know if our neural network could capture fine-grained features beyond human-defined labels. Using cutting-edge animal tracking software SLEAP (Pereira et al., 2022), flies’ wings, heads, tails, and thoraxes were tracked throughout the clip automatically with manual proofreading (Figure 3—video 1). Three straight-forward features were visualized on the t-SNE map. Distances between male heads and female tails could indicate chasing intensity; wing angles of male flies were correlated to wing extension behavior, and distances of male flies away from the chamber center could reflect the trade-off between their thigmotaxis (Besson and Martin, 2005) and courtship motivation. We found that the features extracted by Selfee separated wing extension behaviors based on the wing angles (Figure 3—figure supplement 1C) and male head to female tail distance (Figure 3—figure supplement 1D). The result also showed that chasing behavior could be separated based on the positions of male flies in the chamber, which might indicate the thigmotaxis level. In conclusion, Selfee is capable of extracting comprehensive features that consist of human observations or animal tracking results, and in this way, Selfee uniforms natural-languages-based human descriptions and engineered features from tracking results in different units (e.g., rad for angle and mm for distance) and scales in a single discriminative Meta-representation.

**Figure 3—figure supplement 1.**
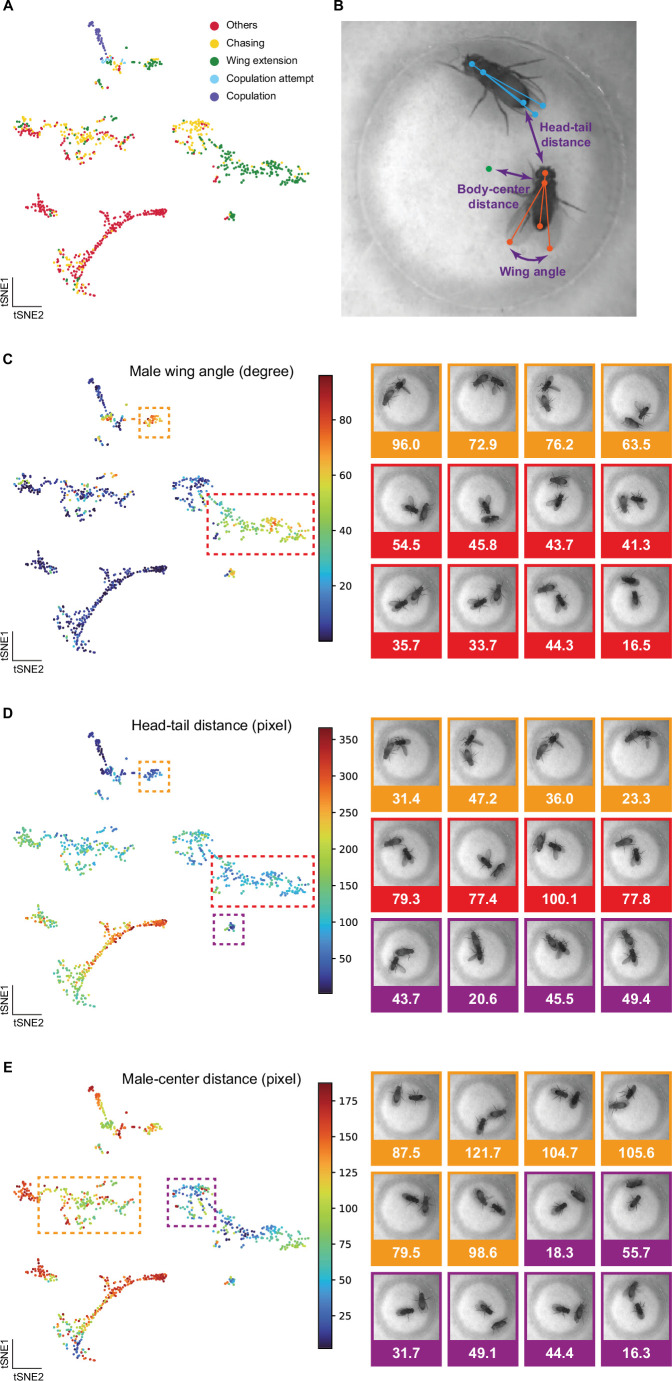
Selfee (**Sel**f-supervised **Fe**atures **E**xtraction) captured fine-grained features related to animal postures and positions. (**A**) Visualization of fly courtship live-frames with t-SNE dimension reduction. Each dot was colored based on human annotations. Points representing non-interactive behaviors (‘others’), chasing, wing extension, copulation attempt, and copulation were colored with red, yellow, green, blue, and violet, respectively. Same as Figure 3A. (**B**) Fly skeletons were semi-automated labeled, and three features were used in the following panels. Five body parts were marked with points, including head, tail, thorax, wings, and head to tail, thorax to wings were linked by lines. Females were indicated by blue color and males were indicated by orange color. Three features were male head to female tail distance, male thorax to chamber center, and the angle between male wings, which were indicated in violet. (**C**) Male wing angles were visualized on the t-SNE map same as panel A. Frames of wing extension behaviors in the red box were of relatively smaller wing angles than those in the orange box. Some examples from these two groups were exhibited on the right, with their angle values below each image. (**D**) Male head to female tail distances were visualized on the t-SNE map same as panel A. Frames of wing extension behaviors in the red box were of relatively shorter distance than those in the orange and violet boxes. Some examples from these three groups were exhibited on the right, with their distance values below each image. (**E**) Male thorax to chamber center distances were visualized on the t-SNE map same as panel A. Frames of chasing behaviors in the violet box were of relatively shorter distance than those in the orange box. Some examples from these two groups were exhibited on the right, with their distance values below each image.

**Figure 3—figure supplement 2.**
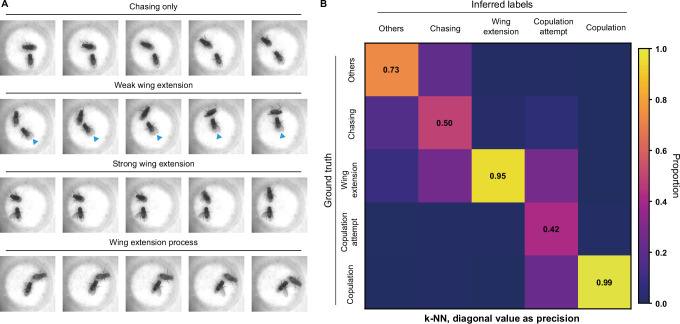
Difficulties on fly courtship behavior classification. (**A**) Some wing extension frames are hard to distinguish from chasing behaviors. Images in the first row were labeled as no wing extension; images in the second to the fourth rows were labeled as wing extension. Images in the second row were of relatively weak wing extension (blue indicators pointed at slightly extended wings), and the fourth row showed a process from no wing extension to strong wing extension. (**B**) The confusion matrix of the *k*-NN classifier, normalized by the numbers of each behavior in inferred labels. The average *F*_1_ score of the sevenfold cross-validation was 72.4%, and mAP was 75.8%. The precision of each class of behaviors was indicated on the diagonal of the confusion matrix.

**Figure 3—figure supplement 3.**
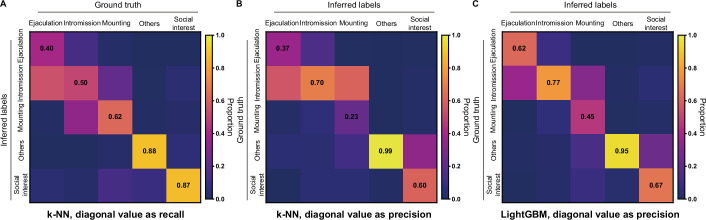
Classification of mice mating behaviors with Selfee (**Sel**f-supervised **Fe**atures **E**xtraction) extracted features. (**A**) For the *k*-NN classifier, the average *F*_1_ score of the eightfold cross-validation was 59.0%, and mAP was 53.0%. The confusion matrix of the *k*-NN classifier, normalized by the numbers of each behavior in the ground truth. The recall of each class of behaviors was indicated on the diagonal of the confusion matrix. (**B**) The confusion matrix of the *k*-NN classifier, normalized by the numbers of each behavior in inferred labels. The precision of each class of behaviors was indicated on the diagonal of the confusion matrix. (**C**) The confusion matrix of the LightGBM (Light Gradient Boosting Machine) classifier, normalized by the numbers of each behavior in inferred labels. The precision of each class of behaviors was indicated on the diagonal of the confusion matrix. The LightGBM classifier had a much better performance compared with the *k*-NN classifier.

**Figure 3—figure supplement 4.**
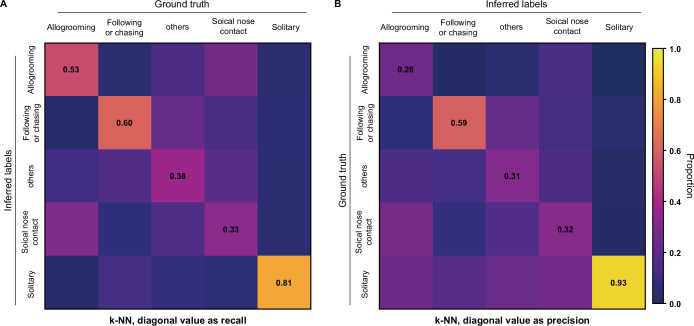
*k*-NN classification of rat behaviors with Selfee (**Sel**f-supervised **Fe**atures **E**xtraction) trained on mice datasets. (**A**) The average *F*_1_ score of the ninefold cross-validation was 49.6%, and mAP was 46.6%. The confusion matrix of the *k*-NN classifier, normalized by the numbers of each behavior in the ground truth. The recall of each class of behaviors was indicated on the diagonal of the confusion matrix. (**B**) The confusion matrix of the *k*-NN classifier, normalized by the numbers of each behavior in inferred labels. The precision of each class of behaviors was indicated on the diagonal of the confusion matrix.

**Figure 3—figure supplement 5.**
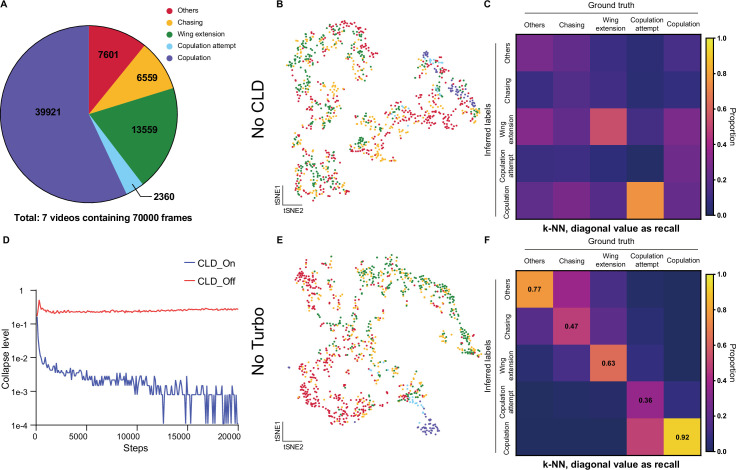
Ablation test of Selfee (**Sel**f-supervised **Fe**atures **E**xtraction) training process on fly datasets. (**A**) The distribution of different behaviors in wild-type flies courtship videos. (**B**) Visualization of the same live-frames as Figure 3A with t-SNE dimension reduction. Used representations were extracted by models trained without cross-level instance-group discrimination (CLD) loss. Each dot is colored based on human annotations. The legend is shared with panel A. (**C**) The confusion matrix of the *k*-NN classifier, normalized by the numbers of each behavior in the ground truth. The recall of each class of behaviors was indicated on the diagonal of the confusion matrix. Used representations were extracted by models trained without CLD loss. (**D**) Collapse levels during the training process. Collapse level was calculated as one minus to the average standard deviation of each channel of the representation multiplied by the square root of the channel number. One means maximum collapse, while zero means no collapse. Without CLD loss, Selfee suffered from catastrophic mode collapse. Details for collapse level calculation could be found in Materials and methods. (**E**) Visualization of the same live-frames as Figure 3A with t-SNE dimension reduction. Used representations were extracted by models trained without Turbo transformation. Each dot is colored based on human annotations. The legend is shared with panel A. (**F**) The confusion matrix of the *k*-NN classifier, normalized by the numbers of each behavior in the ground truth. The recall of each class of behaviors was indicated on the diagonal of the confusion matrix. Used representations were extracted by models trained without Turbo transformation.

**Figure 3—video 1.** Pose estimation of fly courtship behaviors. Flies’ wings, heads, tails, and thoraxes were tracked throughout the clip automatically using cutting-edge animal tracking software SLEAP, and each frame was carefully manual proofread by human researchers.

Meta-representations can also be used for behavior classification. We manually labeled seven 10,000-frame videos (around 5 min each) as a pilot dataset. A weighed *k*-NN classifier was then constructed as previously reported (Wu et al., 2018). Sevenfold cross-validation was performed on the dataset with the *k*-NN classifier, which achieved a mean *F*_1_ score of 72.4% and achieved a similar classification result as human annotations (Figure 3B and C). The classifier had the worst recall score on wing extension behaviors (67% recall, Figure 3B), likely because of the ambiguous intermediate states between chasing and wing extension (Figure 3—figure supplement 2A). The precisions also showed that this *k*-NN classifier tended to have strict criteria for wing extension and copulation and relatively loose criteria for chasing and copulation attempts (Figure 3—figure supplement 2B). It was reported that independent human experts could only reach agreements on around 70% of wing extension frames (Leng et al., 2020), comparable to the performance of our *k*-NN classifier.

Next, we compared Selfee extracted features with animal-tracking derived features. In a previous study Leng et al., 2020, labeled wing extension intensities of male flies for three clips of male-female interaction videos: each frame was scored from 0 (no wing extension) to 3 (strong wing extension) by two experienced researchers. Here, we used their summarized score as ground-truth labels. FlyTracker (Fleet et al., 2014) was used to track each fly’s body, wings and legs, and the identity swap between male and female flies was manually corrected in the previous work. We used four types of post-processing for the tracking results. Features from FlyTracker and JAABA (Kabra et al., 2013) were from the work by Leng et al., 2020. We also constructed pure distance-based features recording distances between all key points (heads, tails, thoraxes, wings, and with or without legs). For a fair comparison, we evaluated the performance of weight *k*-NN classifiers in sixfold cross-validations for all types of features, and none of the additional temporal information aggregation was used (e.g., sliding window voting used in Selfee, bout features used in FlyTracker, or window features used in JAABA). Three evaluation metrics were applied, including a robust version of Pearson’s correlation coefficient (Lai et al., 2019), *F*_1_ score, and average precision. We found that Selfee extracted features achieved comparable results with FlyTracker features or JAABA features, and it achieved the best performance evaluated by Pearson’s correlation and *F*_1_ score (Table 1). We also found that additional irrelevant key points marking fly legs would strongly interfere the performance of pure distance-based features, indicating that key point choice and feature engineering were crucial for downstream classification. The comparison also yielded an interesting result that the pure distance-based feature could even outperform human-engineered features like FlyTracker features. Distances between key points indeed preserved detailed information on animal behaviors, but it remained unclear if they could capture the universals of behavioral stereotypes. For further investigation, we visualized distance-based features and human-engineered features on the same clip as in Figure 3A with t-SNE dimension reduction. Results showed that distance-based features were overfocused on subtle differences between frames and neglected differences between major types of behaviors. In contrast, human-engineered features formed distinct clusters corresponding to human annotations on the t-SNE map (Figure 1—figure supplement 2), so did Selfee features (Figure 3A). Therefore, although pure distance-based features could outperform human-engineered features and Selfee features using highly non-linear *k*-NN classifier, they were less abstractive. Overall, Selfee extracted features are as discriminative as classic animal-tracking derived features, but could be used more easily without careful key points definition or dedicated feature engineering.

**Table 1.** A comparison between Selfee (**Sel**f-supervised **Fe**atures **E**xtraction) extracted features and animal-tracking derived features.

Evaluationssetups	Pearson’s R	*F*_1_ score	AP
Selfee	**0.774** ^*^	**0.629** ^*^	0.354
FlyTracker ->FlyTracker	0.756	0.571	0.330
FlyTracker ->JAABA	0.755	0.613	0.346
FlyTracker (w/o legs) ->distance	0.771	0.613	**0.374** *
FlyTracker (w/ legs) ->distance	0.629	0.400	0.256

* Best results of different feature extractors under each evaluation metric are indicated in bold values.

We then asked whether Selfee can be generalized to analyze behaviors of other species. We fine-tuned fly video pre-trained Selfee with mice mating behavior data. The mating behavior of mice can be defined mainly into five categories (McGill, 1962), including social interest, mounting, intromission, ejaculation, and others (see Materials and methods for detailed definitions). With t-SNE visualization, we found that five types of behaviors could be separated by Selfee, although mounting behaviors were rare and not concentrated (Figure 3D). We then used eight human-annotated videos to test the *k*-NN classification performance of Selfee-generated features. We achieved an *F*_1_ score of 59.0% (Table 3-Replication 1, Figure 3—figure supplement 3). Mounting, intromission, and ejaculation share similar static characteristics but are different in temporal dynamics. Therefore, we asked if more temporal information would assist the classification. Using the LightGBM (Light Gradient Boosting Machine) classifier (Ke et al., 2017), we achieved a much higher classification performance by incorporating slide moving average and standard deviation of 81-frame time windows, the main frequencies, and their energy within 81-frame time windows. The average *F*_1_ score of eightfold cross-validation could reach 67.4%, and the classification results of the ensembled classifier (see Materials and methods) were closed to human observations (Figure 3E and F). Nevertheless, it was still difficult to distinguish between mounting, intromission, and ejaculation because mounting and ejaculation are much rarer than social body contact or intromission.

Selfee is more robust than the vanilla SimSiam networks when applied to the behavioral data. Behavioral data often suffer from severe imbalance. For example, copulation attempts are around sixfold rarer than wing extension during fly courtship (Figure 3—figure supplement 5A). Therefore, we added group discriminators to vanilla SimSiam networks, which were reported to fight against the long-tail effect proficiently (Wang et al., 2021). As noted in the original publication of CLD loss, *k*-means clustering lifted weights of rare behaviors in batches from the reciprocal of the batch size to the reciprocal of the cluster number, for which the imbalance between majority and minority classes can be ameliorated (Wang et al., 2021). Aside from overcoming the long-tail effect, we also found group discriminators helpful for preventing mode collapse during ablation studies (Figure 3—figure supplement 5B-D and Table 2). We hypothesized that the convergence could be easily reached on images of similar objects (two flies), by which CNNs may not be well trained to extract good representations. Without CLD loss, it could be easier to output similar representations than to distinguish images apart, because only the attraction between positive samples was applied. Using CLD loss, Selfee could explicitly utilize negative samples and encourage both repulsion and attraction. Therefore, the mode collapse could be largely avoided. Aside from CLD loss, Turbo transformation was another customed modification. Applying the Turbo lookup table on grayscale frames brought more complexity and made color distortions more powerful on grayscale images, one of the most critical type of augmentations reported before (Chen et al., 2020). Because hue and saturation of a grayscale image are meaningless, color jitter can be strongly enhanced after Turbo transforms. Selfee would capture more useful features with this Turbo augmentation (Figure 3—figure supplement 5E, F and Table 2). Live-frames used by Selfee were also helpful to capture temporal information of animal behaviors, especially for highly dynamic ones such as mice mating behaviors. We found that Selfee features extracted from single frames were less discriminative than those extracted from live-frames (Table 3). In summary, our modifications to original SimSiam designs, including live-frames extracting temporal dynamics, Turbo transformation customed for grayscale images, and CLD loss coping with the long-tailed nature of behavioral data, provide a significant performance boost in the case of animal behavior analysis.

**Table 2.** An ablation test of Selfee (**Sel**f-supervised **Fe**atures **E**xtraction) training process on fly datasets.

Model	Pre-trained ResNet-50 with random projectors		Selfee		Selfee without CLD loss		Selfee without Turbo transformation	
Evaluation	Mean *F*_1_ score	Mean AP	Mean *F*_1_ score	Mean AP	Mean *F*_1_ score	Mean AP	Mean *F*_1_ score	Mean AP
Replication 1	0.586	0.580	0.724	0.758	0.227	0.227	0.604	0.550
Replication 2	0.597	0.570	0.676	0.683	0.163	0.200	0.574	0.551
Replication 3	0.596	0.586	0.714	0.754	0.172	0.214	0.517	0.497
Best	0.597	0.586	**0.724** ^*^	**0.758** ^*^	0.227	0.227	0.604	0.551

* Best results of different training setups under each evaluation metric are indicated in bold values.

**Table 3.** An ablation test of Selfee training process on mice datasets.

Model	Single frame + KNN		Live-frame + KNN		Single frame + LGBM		Live-frame + LGBM	
Evaluation	Mean *F*_1_ score	Mean AP	Mean *F*_1_ score	Mean AP	Mean *F*_1_ score	Mean AP	Mean *F*_1_ score	Mean AP
Replication 1	0.554	0.498	0.590	0.530	0.645	0.671	0.674	0.691
Replication 2	0.574	0.508	0.599	0.549	0.653	0.663	0.663	0.699
Replication 3	0.566	0.514	0.601	0.539	0.652	0.692	0.663	0.700
Mean	0.565	0.507	0.597	0.539	0.650	0.675	**0.667** ^*^	**0.697** ^*^
Best	0.574	0.514	0.601	0.549	0.653	0.692	**0.674** ^*^	**0.700** ^*^

* Best results of different training setups under each evaluation metric are indicated in bold values.

### Anomaly detection at the frame level identifies rare behaviors at the sub-second time scale

**Figure 4.**
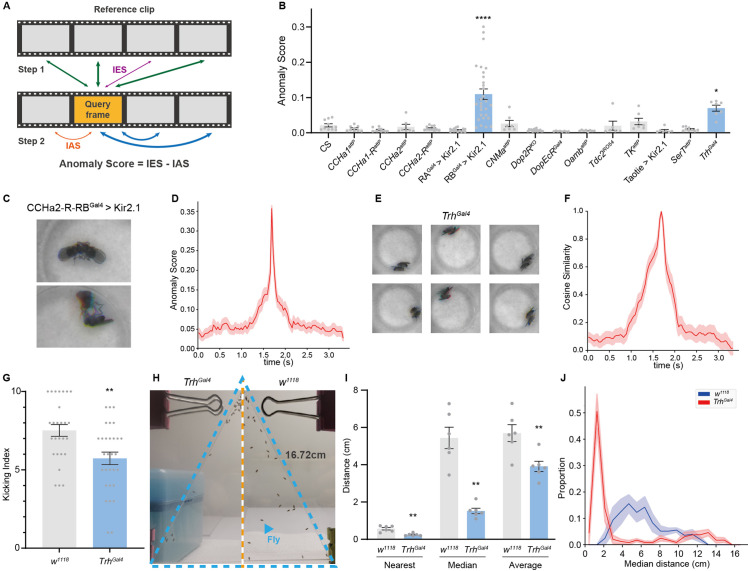
Anomalous posture detection using Selfee (**Sel**f-supervised **Fe**atures **E**xtraction)-produced features. (**A**) The calculation process of anomaly scores. Each query frame is compared with every reference frame, and the nearest distance was named IES (the thickness of lines indicates distances). Each query frame is also compared with every query frame, and the nearest distance is called IAS. The final anomaly score of each frame equals IES minus IAS. (**B**) Anomaly detection results of 15 fly lines with mutations in neurotransmitter genes or with specific neurons silenced ( n = 10,9,10,7,12,15,29,7,16,8,8,6,7,7,9,7, respectively). RA is short for CCHa2-R-RA, and RB is short for CCHa2-R-RB. CCHa2-R-RB^Gal4^>Kir2.1, q<0.0001; *Trh^Gal4^*, q=0.0432; one-way ANOVA with Benjamini and Hochberg correction. (**C**) Examples of mixed tussles and copulation attempts identified in CCHa2-R-RB^Gal4^>Kir2.1 flies. (**D**) The temporal dynamic of anomaly scores during the mixed behavior, centralized at 1.67 s. SEM is indicated with the light color region. (**E**) Examples of close body contact behaviors identified in *Trh^Gal4^* flies. (**F**) The cosine similarity between the center frame of the close body contact behaviors (1.67 s) and their local frames. SEM is indicated with the light color region. (**G**) The kicking index of *Trh^Gal4^* flies (*n*=30) was significantly lower than *w^1118^* flies (*n*=27), p=0.0034, Mann-Whitney test. (**H**) Examples of social aggregation behaviors of *Trh^Gal4^* flies and *w^1118^* flies. Forty male flies were transferred into a vertically placed triangle chamber (blue dashed lines), and the photo was taken after 20 min. A fly was indicated by a blue arrow. The lateral sides of the chamber were 16.72 cm. (**I**) Social distances of *Trh^Gal4^* flies (*n*=6) and *w^1118^* flies (*n*=6). *Trh^Gal4^* flies had much closer social distances with each other compared with *w^1118^* flies; nearest, p=0.0043; median, p=0.002; average, p=0.0087; all Mann-Whitney test. (**J**) Distributions of the median social distance of *Trh^Gal4^* flies and *w^1118^* flies. Distributions were calculated within each replication. Average distributions were indicated with solid lines, and SEMs were indicated with light color regions.

The representations produced by Selfee could be directly used for anomaly detection without further post-processing. During the training step, Selfee learns to compare Meta-representations of frames with cosine distance which is also used for anomaly detection. When given two groups of videos, namely the query group and the reference group, the anomaly score of each live-frame in the query group is calculated in two steps (Figure 4A). First, distances between the query live-frame and all reference live-frames are measured, and the *k*-nearest distance is referred to as its inter-group score (IES). Without further specification, *k* equals 1 in all anomaly detections in this work, which is a trivial and intuitive case of the *k*-NN algorithm to avoid parameter search of *k*. Some false positives occurred when only the IES was used as the anomaly score (Figure 4—figure supplement 1A). The reason could be that two flies in a chamber could be in mathematically infinite relative positions and form a vast event space. However, each group usually only contains several videos, and each video is only recorded for several minutes. For some rare postures, even though the probability of observing them is similar in both the query and reference group, they might only occur in the query group but not in the reference group. Therefore, an intra-group score (IAS) is introduced in the second step to eliminate these false-positive effects. We assume that those rare events should not be sampled frequently in the query groups either. Thus, the IAS is defined as the *k*-nearest distance of the query frame against all other frames within its group, except those within the time window of ±50 frames, because representations for frames beyond 50 frames were less similar to the current frame (Figure 4—figure supplement 1B). The final anomaly score is defined as the IES minus the IAS.

**Figure 4—figure supplement 1.**
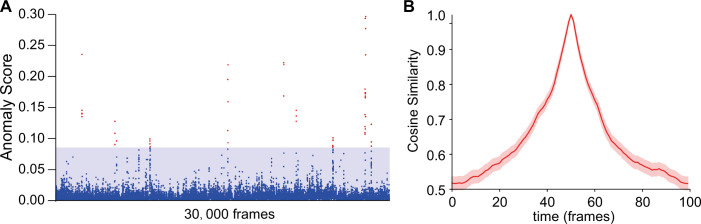
Using intra-group score (IAS) to eliminate false-positive results in anomaly detections. (**A**) Anomaly scores without IAS of wild-type male-male interactions with the same genotype as references. The blue region indicates the max anomaly score when using IAS; blue dots indicate anomaly scores without IAS that fall into the blue region; red dots indicate false-positive anomaly scores. (**B**) The cosine similarity between the center frame of wild-type courtship behaviors (1.67 s) and their local frames. SEM is indicated with the light color region. Seven videos containing 70,000 frames were split into non-overlapping 100-frame fragments for calculations. Beyond ±50 frames, the cosine similarity dropped to a much lower level, not affecting anomaly detection.

**Figure 4—video 1.** The anomaly detection on male-male interactions of RB-Gal4 >Kir2.1 flies. The anomalous behavior was brief tussle behavior mixed with copulation attempts. This behavior was ultra-fast and lasted for less than a quarter second.

**Figure 4—video 2.** The anomaly detection on male-male interactions of *Trh^Gal4^* flies. The anomalous behavior was short-range body interactions. These social interactions could last for around half to 1 s on average.

To test whether our methods could detect anomalous behavior in real-world data, we performed anomaly detection to 15 previously recorded neurotransmitter-related mutant alleles or neuron-silenced lines (with UAS-Kir2.1; Paradis et al., 2001; Figure 4B). Their male-male interaction videos were inferred by Selfee trained on male-female courtship videos. Since we aimed to find interactions distinct from male-male courtship behaviors, a baseline of ppk23 >Kir2.1 flies was established because this line exhibits strong male-male courtship behaviors (Thistle et al., 2012). We compared the top-100 anomaly scores from sets of videos from experimental groups and wild-type control flies. The results revealed that one line, CCHa2-R-RB>Kir2.1, showed a significantly high anomaly score. By manually going through all anomalous live-frames, we further identified its phenotype as a brief tussle behavior mixed with copulation attempts (Figure 4C, Figure 4—video 1, 0.2× play speed). This behavior was ultra-fast and lasted for less than a quarter second (Figure 4D), making it difficult to be detected by human observers. Up to this point, we have demonstrated that the frame-level anomaly detection could capture sub-second behavior episodes that human observers tend to neglect.

Selfee also revealed that *Trh* (*Tryptophan hydroxylase*) knock-out flies had close body contact compared to wild-type. *Trh* is the crucial enzyme for serotonin biosynthesis (Coleman and Neckameyer, 2005), and its mutant flies showed a statistically significantly higher anomaly score (Figure 4B) than the wild-type control. Selfee identified 60 frames of abnormal behaviors within 42,000 input frames, occupying less than 0.15% of the total recording time. By manually going through all these frames, we concluded most of them as short-range body interactions (Figure 4E and Figure 4—video 2, 0.2× play speed). These social interactions could last for around 1 s on average (Figure 4F). Even though serotonin signals were well studied for controlling aggression behavior in flies (Alekseyenko et al., 2014), to the best of our knowledge, the close body contact of flies and serotonergic neurons’ role in this behavior has not been reported yet. Considering this behavior is not as fast as the ones of CCHa2-R-RB>Kir2.1 flies for humans, a possible reason is that this behavior is too scarce to be noticed by human experts.

To further ask whether these close body contacts have biological significance, we performed corresponded behavior assays on mutant flies. Based on the fact that the *Trh* mutant male flies have a higher tolerance to body touch, we hypothesized that they would have a decreased defensive behavior. As previously reported, fruit flies show robust defensive behavior to mechanical stimuli on their wings (Li et al., 2016; Liu et al., 2020). Decapitated flies would kick with their hind legs when a thin probe stimulates their wings. This stimulation mimics the invasion of parasitic mites and could be used to test its defensive behavior. Our results showed that *Trh* knock-out flies had a significantly lower kicking rate than control flies (Figure 4G), indicating a reduction of self-defensive intensity. Next, we performed social behavior assay (McNeil et al., 2015; Simon et al., 2012) on the mutant flies because the close body contact can also be explained by reduced social distance. We measured the nearest distance, median distance, and average distance of each male fly in a 40-individual group placed in a vertical triangular chamber (Figure 4H). By comparing median values of these distances of each replication, *Trh* knock-out flies kept significantly shorter distances from others than the control group (Figure 4H, I). The probability density function of their median distances also showed that knock-out flies had a closer social distance than control flies (Figure 4J). Therefore, we concluded that *Trh* knock-out flies had reduced self-defensive behavior and social distance, which validated the anomaly detected by Selfee. Taken together, Selfee is capable of discovering novel features of animal behaviors with biological relevance when a proper baseline is defined.

### Modeling motion structure of animal behaviors

**Figure 5.**
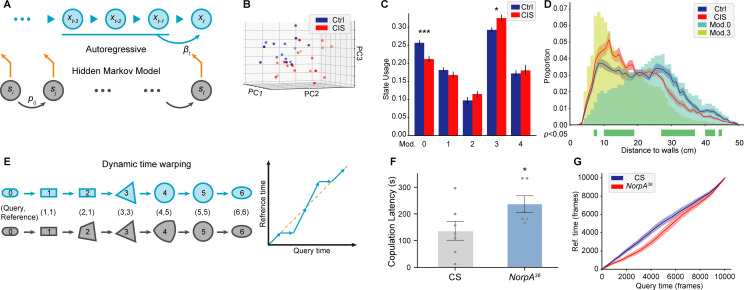
Time-series analyses using Selfee (**Sel**f-supervised **Fe**atures **E**xtraction)-produced features. (**A**) A brief illustration of the autoregressive hidden Markov model (AR-HMM). The local autoregressive property is determined by *β_t_*, the autoregressive matrix, which is yield based on the current hidden state of the HMM. The transition between each hidden state is described by the transition matrix (*p_ij_*). (**B**) Principal component analysis (PCA) visualization of state usages of mice in control groups (*n*=17, blue points) and chronic immobilization stress (CIS) groups (*n*=17, red points). (**C**) State usages of 10 modules. Module No.0 and No.3 showed significantly different usages in wild-type and mutant flies; p=0.00065, q=0.003 and p=0.015, q=0.038, respectively, Mann-Whitney test with Benjamini and Hochberg correction. (**D**) The differences spotted by the AR-HMM could be explained by the mice’s position. Mice distances to the two nearest walls were calculated in each frame. Distance distributions (the bin width was 1 cm) throughout open-field test (OFT) experiments were plotted in solid lines, and SEMs were indicated with light color regions. Green blocks indicated bins with statistic differences between the CIS group and control groups. Frames assigned to modules No.0 and No.3 were isolated, and their distance distributions were plotted in blue and yellow bars, respectively. Frames of module No.0 were enriched in bins of larger values, while frames of module No.3 were enriched in bins of smaller values. (**E**) A brief illustration of the dynamic time warping (DTW) model. The transformation from a rounded rectangle to an ellipse could contain six steps (gray reference shapes). The query transformation lags at step 2 but surpasses at step 4. The dynamic is visualized on the right panel. (**F**) *NorpA^36^* flies (*n*=6) showed a significantly longer copulation latency than wild-type flies (*n*=7), p=0.0495, Mann-Whitney test. (**G**) *NorpA^36^* flies had delayed courtship dynamics than wild-type flies with DTW visualization. Dynamic of wild-type flies and *NorpA* mutant flies were indicated by blue and red lines, respectively, and SEMs were indicated with light color regions. The red line was laid below the blue line, showing a delayed dynamic of *NorpA* mutant flies.

Animal behaviors have long-term structures beyond single-frame postures. The duration and proportions of each bout and transition probabilities of different behaviors have been proven to have biological significance (Mueller et al., 2019; Wiltschko et al., 2015). To better understand those long-term characteristics, we introduce AR-HMM and DTW analyses to model the temporal structure of animal behaviors. AR-HMM is a powerful method for analyzing stereotyped behavioral data (Rudolph et al., 2020; Wiltschko et al., 2015; Wiltschko et al., 2020). It discovers modules of behaviors and describes the modules with autoregressive matrixes. In other words, each embedding of the frame is predicted by a linear combination of embeddings of several previous frames, and frames within each module share the same coefficients of the linear combination, which is called autoregressive matrixes. In an AR-HMM, these fitted autoregressive patterns are utilized as hidden states of the HMM. The transition between each hidden state (autoregressive pattern) is determined by the transition matrix of the HMM. By the definition of Markov property, the transition from the current state to the next state is only determined by the current state and transition probabilities (Figure 5A). In this way, AR-HMM could capture local structures (autoregressive pattern) of animal behaviors as well as syntaxes (transition probabilities).

**Figure 5—video 1.** A video example of Module No.0. An exploratory-like behavior of mice.

**Figure 5—video 2.** A video example of Module No.3. Mice walking alongside walls of the arena.

We asked if we could detect the dynamic changes in mice behaviors after chronic immobilization stress (CIS) during the open-field test (OFT). The CIS model is well established to study the depression-like behavior of experimental animals, and the anxiety level of mice can be evaluated with OFT. Mice prefer to walk near the wall, and the time spent in the center of the arena is considered to be related with anxiety behavior (Crusio et al., 2013; Prut and Belzung, 2003). We tested the OFT performance of mice with or without the CIS treatment. After preprocessing, videos were processed with Selfee trained with mice mating behavior. An AR-HMM with five modules (No.0 to No.4) was fitted to analyze behaviors during OFT. PCA of state usages revealed an apparent difference between mice with and without CIS experience (Figure 5B). Usages of two modules (No.0 and No.3) showed statistically significant differences between the two groups (Figure 5C). By watching sampled fragments of these two behaviors, we found module No.0 might be exploratory-like behavior, while module No.3 contained mice walking alongside walls (Figure 5—videos 1 and 2). To further confirm these observations, videos were analyzed by an animal tracking program, whose results were proofread manually. Mice in the CIS group spent more time near walls while mice in the control group spent more time in the central area (Figure 5C, blue and red lines), and similar observations had been reported before (Ramirez et al., 2015). Then, we analyzed whether these two modules were related to mice’s position in the arena. All frames belonging to each module were extracted, and distances to the two nearest walls were calculated, the same as what was performed on all videos. The result indicated that mice performing behavior No.0 were relatively distant from walls, while in module No. 3, mice were closer to the border (Figure 5C, cyan and yellow bars). These results showed that Selfee with AR-HMM successfully distinguished mice in the control group from the CIS group, and the differences spotted by Selfee were consistent with previous observations (Ramirez et al., 2015). It is also established that Selfee with AR-HMM could discover the differences in proportions of behaviors, similar to what could be achieved with classic manual analysis or animal tracking software.

The AR-HMM modeling does not necessarily capture the difference in long-term dynamics intuitively, such as the latency of certain behaviors. To solve this problem, we introduce DTW analysis. DTW is a well-known algorithm for aligning time series, which returns the best-matched path and the matching similarity (Figure 5E). The alignment can be simplified as follows. When given the same start state and end state, it optimally maps all indices from the query series to the reference series monotonically. Pairs of mapped indices form a path to visualize the dynamic difference. The points above the diagonal line indicate that the current time point in the query group is matched to a future time point in the reference group so that the query group has faster dynamics and vice versa. Our experiments use cosine similarities of Selfee extracted representations to calculate warping paths.

Previously, DTW was widely applied to numerical measures of animal behaviors, including trajectory (Cleasby et al., 2019), audios (Kohlsdorf et al., 2016), and acceleration (Aurasopon, 2016). For the first time, we applied DTW to image data, with the aid of Selfee, to study the prolonged dynamic of animal behaviors. We applied DTW to analyze representations of *NorpA* mutant flies. Visual cues are essential for male flies to locate female flies during courtship (Ribeiro et al., 2018), and mutant flies of *NorpA,* which have defective visual transduction (Bloomquist et al., 1988), have a prolonged courtship latency in our experiments (Figure 5F), similar to previously findings (Markow and Manning, 1980). When wild-type flies were used as the reference for the DTW, the group of *NorpA* mutant flies yielded a curve lower than the diagonal line, indicating a delay in their courtship behaviors (Figure 5G). In this way, our experiments confirm that Selfee and DTW could capture differences in long-term dynamics such as behavior latency. In conclusion, DTW and AR-HMM could capture temporal differences between control and experimental groups beyond single-frame postures, making Selfee a competent unsupervised method for traditional analyses like courtship index or copulation latency.

### A brief demonstration of the whole Selfee pipeline

**Figure 6.**
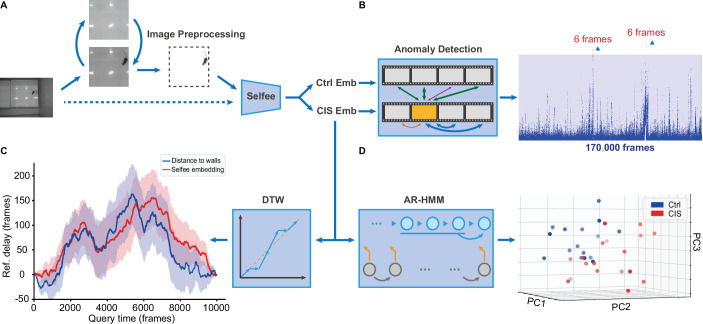
Application of the Selfee (**Sel**f-supervised **Fe**atures **E**xtraction) pipeline to mice open-field test (OFT) videos. (**A**) Image preprocessing for Selfee. The area of the behavior chamber was cropped, and the background was extracted. Illumination normalization was performed after background subtraction. This preprocessing could be skipped if the background was consistent in each video, as our pipeline for fly videos (dashed lines). (**B**) Anomaly detection of mice OFT videos after chronic immobilization stress (CIS) experiences. Only 12 frames (red points, indicated by arrows) were detected based on a threshold constructed with control mice (the blue region), and anomaly scores were slightly higher than the threshold. (**C**) Dynamic time warping (DTW) analysis of mice OFT videos after CIS experiences. The dynamic difference between control groups and CIS groups was visualized, and positive values indicated a delay of the reference (control groups). Results from Selfee features and animal positions were similar (red and blue lines, respectively). (**D**) Autoregressive hidden Markov model (AR-HMM) analysis of mice OFT videos after CIS experiences. Principal component analysis (PCA) visualization of state usages of mice in control groups (*n*=17, blue points) and CIS groups (*n*=17, red points). Same as Figure 5B.

Selfee is a powerful end-to-end unsupervised behavior analysis tool. We went through the whole pipeline using the mice OFT discussed in previous sections as a demonstration. The first step to use Selfee is setting up a development environment containing packages in Key resources table. When recording conditions are consistent for different sets of videos, preprocessing can be simple frame extraction (Figure 6A, the dashed line). As mice open-field videos were recorded with some variations in our case, arenas in the video were first cropped; backgrounds were removed, and the luminance was normalized (Figure 6A, solid lines).

**Figure 6—figure supplement 1.**
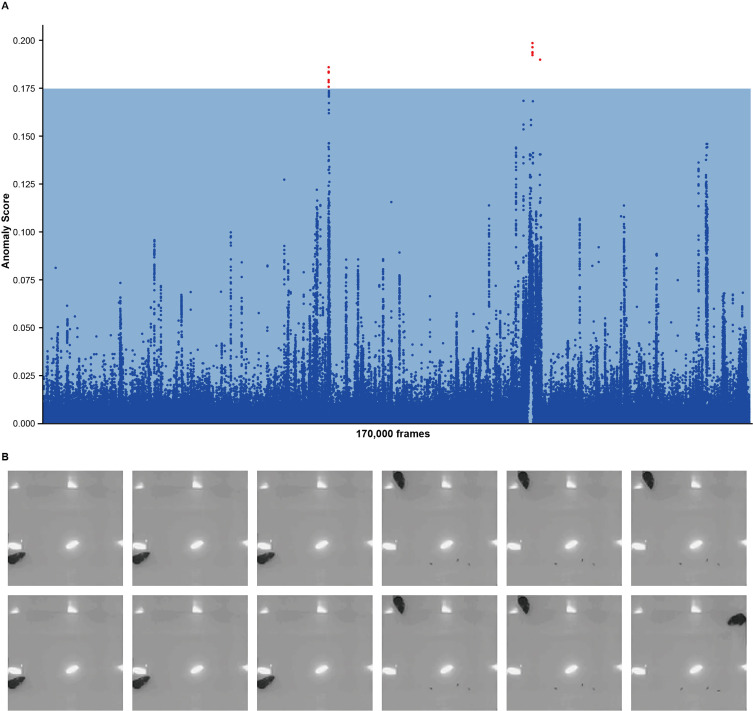
Anomaly detection of chronic immobilization stress (CIS) mice. (**A**) Anomaly scores of CIS mice with half of mice in the control groups as references and another half as negative controls. The blue region indicates the max anomaly score of negative controls; blue dots indicate anomaly scores fall into the blue region; red dots indicate detected anomalies (12 points in total). Detected anomalies were only of scores slightly higher than the threshold. (**B**) Twelve anomalous frames. These frames contained mice near walls with their head facing the center of the arena. All frames seemed normal.

**Figure 6—figure supplement 2.**
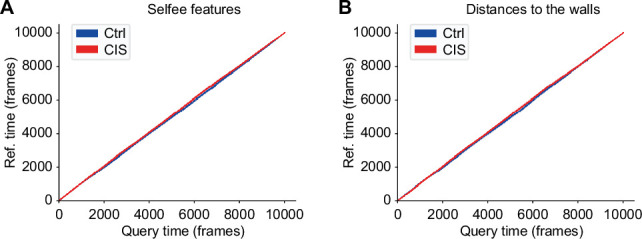
Dynamic time warping (DTW) analysis of chronic immobilization stress (CIS) and control mice. (**A**) DTW analysis using Selfee (**Sel**f-supervised **Fe**atures **E**xtraction) features of mice in CIS groups with control mice as reference. The red line represents CIS groups, and the blue line represents control groups. The red line is slightly above the blue line. (**B**) DTW analysis using mice distances to two nearest walls of mice in CIS groups with control mice as reference. The red line represents CIS groups, and the blue line represents control groups. The red line is slightly above the blue line.

After preprocessing, image embeddings were extracted with pre-trained Selfee. Features were then grouped according to experimental designs, and features for mice in the control group and CIS group were sent to the following modules. First, features of the control group were randomly assigned as references or negative controls. The maximum anomaly score of negative controls was set as the threshold. In the CIS group, 12 frames of anomaly behaviors were sorted out (Figure 6A, Figure 6—figure supplement 1A). However, these anomaly frames with their score slightly higher than the threshold only contributed to less than 0.01% of all frames, and occurred only in 2 out of 17 videos. Therefore, these frames were classified as false-positive after being examined manually (Figure 6—figure supplement 1B). Second, the features of the control group and CIS group were analyzed with AR-HMM. As previously showed, AR-HMM separated two groups apart, and the major differences were related to mice’s distances to the arena walls (Figure 5D).

Finally, we compared the long-range dynamics of these two groups of mice with DTW. DTW results showed a relatively similar dynamic between the two groups, with a minor delay occurring in the control group (Figure 6—figure supplement 2A). Inspired by the result of AR-HMM, we wondered if the delay could also be explained by the distance to walls. Therefore, DTW analysis was applied to mice distances to the arena walls, and the result appeared similar (Figure 6—figure supplement 2B). When delays of the control group were isolated, we found results generated from Selfee embeddings were strongly consistent with those from mice positions (Figure 6C). Although the observed delay was related to mice positions in the arena, it was only several seconds, and the biological significance was unclear. Further experiments were required to determine whether this result indeed revealed the behavioral dynamic of mice with CIS experiences, or just a deviation due to small sample sizes. In conclusion, our pipeline with Selfee and downstream analysis could evaluate animal behaviors in a purely unsupervised way. The method requires no human definition of either animal behaviors, or key points of animal skeletons, and thus subjective factors that would bias the analysis are avoided.

## Discussion

Here, we use cutting-edge self-supervised learning methods and CNNs to extract Meta-representations from animal behavior videos. Siamese CNNs have proven their capability to learn comprehensive representations (Chen et al., 2020). The cosine similarity, part of its loss function used for training, is rational and well suited to measure similarities between the raw images. Besides, CNNs are trained end-to-end so that preprocessing steps like segmentation or key points extraction is unnecessary. By incorporating Selfee with different post-processing methods, we can identify phenotypes of animal behaviors at different time scales. In the current work, we demonstrate that the extracted representations could be used not only for straightforward distance-based analyses such as t-SNE visualization or *k*-NN anomaly detection but also for sophisticated post-processing methods like AR-HMM. These validations confirm that the extracted Meta-representations are meaningful and valuable. Besides anomaly detection, AR-HMM, and DTW discussed here, other methods could also be used to process features produced by Selfee. For classification, as mentioned before, temporal features could be engineered, such as bout features used in FlyTracker, or window features used in JAABA. Also, other unsupervised learning methods developed for skeleton features can be used downstream of Selfee. For example, UMAP non-linear transformations (McInnes et al., 2018) and HDBSCAN (Campello et al., 2013) clustering proposed in B-SOiD (Hsu and Yttri, 2021) and dynamic time alignment kernel proposed in Behavior Atlas (Huang et al., 2021). Therefore, Selfee features could be used flexibly with different machine learning methods for various experimental purposes.

By applying our method to mice mating behavior and fly courtship behaviors, we show that Selfee could serve as a helpful complement of the widely used pose estimation methods in multi-animal behavior analysis, and vice versa. First, Selfee features are proved to be comparably discriminative as key points derived human-engineered features. Second, the famous DeepLabCut (Lauer et al., 2021; Mathis et al., 2018) and similar methods face problems coping with animals of the same color recorded at a compromised resolution and with intensive body contacts. We found that the current version of DeepLabCut could hardly extract useful features during intromission behaviors of two black mice (Figure 1—figure supplement 3A, Figure 1—video 1). The reason was that it was extremely difficult to unambiguously label body parts like nose, ears and hips when two mice were close enough, a task challenging even for human experts. Similar results were also observed in fly videos. We visualized tracking results provided by the Fly-vs-Fly dataset (Fleet et al., 2014), which used classic computer vision techniques for tracking, and inaccurate tracking of fly wings and fly bodies was observed (Figure 1—figure supplement 3B, Figure 1—video 2). To investigate if cutting-edge deep learning methods would avoid such problems, we labeled 3000 frames to train a SLEAP (Pereira et al., 2022) model and inferred on a clip of a fly courtship video. Unfortunately, the same type of error occurred when flies showed intensive body contact (Figure 1—figure supplement 3C, Figure 1—video 3). Those wrongly tracked frames were either hard for humans to detect or rare postures that were not covered in the training data. By testing these three representative pose estimation programs, we argue that key point detection for closely interacting or overlapped animals is still challenging. By contrast, our methods could capture global characteristics of behaviors like human perception, making it robust to these confusing occlusions. Nevertheless, Selfee features appear less explainable than key points, and we have shown that animal tracking was very useful for interpreting Selfee features. Therefore, Selfee strongly complements the incapability of pose estimation methods processing closely contacted animals, and could be further improved to be more explainable when assisted by animal tracking.

We also demonstrate that the cutting-edge self-supervised learning model is accessible to biology labs. Modern self-supervised learning neural networks usually require at least eight modern GPUs (Caron et al., 2021; Wang et al., 2021) even TPUs (Chen et al., 2020; Grill et al., 2020) for training, take advantage of batch sizes larger than 1024, and training time varies from several days to a week. In contrast, our model can be trained on only one RTX 3090 GPU with a batch size of 256 within only 8 hr with the help of the newly proposed CLD loss function (Wang et al., 2021) and other improvements (see Materials and methods for further details). Despite our effort to improve training speed and lower hardware requirement, the current development of self-supervised learning could not make training as accessible as cutting-edge supervised key points detection networks. Nevertheless, we found that our model could achieve zero-shot domain transfer. We demonstrated that Selfee trained for mating behavior of a pair of mice could also be applied to OFTs of single animal. Assisted by AR-HMM, Selfee captured the major differences between mice in the control and CIS groups. Even though the network was trained in a translation-invariant way, and apparent landmarks in the arena were removed during preprocessing, Selfee still identified two distinct behaviors related to mice position in the arena in a zero-shot way. Furthermore, when the model pre-trained with mice videos was applied to rat behaviors, we were able to achieve a zero-shot classification of five major types of social behaviors (Figure 3—figure supplement 4). Although the *F*_1_ score was only 49.6%, it still captured the major differences between similar behaviors, such as allogrooming and social nose contact (Figure 3—figure supplement 4). These results showed that Selfee could be used in a zero-shot training-free way even without GPUs. Thus, we have demonstrated that self-supervised learning could be easily achieved with limited computation resources and a much shorter time and could be directly transferred to datasets that share similar visual characteristics and save more resources.

Despite those advantages, there are some limitations of Selfee. First, because each live-frame only contains three raw frames, our model could not capture much information on the animal motion. It becomes more evident when Selfee is applied to highly dynamic behaviors such as mice mating behaviors. This can be overcome with updated hardware because 3D convolution (Ji et al., 2013) or spatial-temporal attention (Aksan et al., 2020) is good at dynamic information extraction but requires much more computational resources. Second, as previously reported, CNNs are highly vulnerable to image texture (Geirhos et al., 2019). We observed that certain types of beddings of the behavior chamber could profoundly affect the performance of our neural networks (Figure 1—figure supplement 1), so in some cases, background removal is necessary (see Materials and methods for further details). Lastly, Selfee could only use discriminative features within each batch, without any negative samples provided, so minor irrelevant differences could be amplified and cause inconsistent results (named mode-split). This mode-split may increase variations of downstream analyses. One possible solution is using some labels to fine-tune the network (cite instance learning). However, the fine-tuning would break the fully unsupervised setup. Another solution is to make the representations more explainable, so that the causes of mode-split can be spotted and corrected. We propose that by combining unsupervised semantic segmentation (Cho et al., 2021; Hamilton et al., 2022; Xu et al., 2022) and bag-of-features (BagNet) (Brendel and Bethge, 2019) training objective, it is possible to produce disentangle features that could map each dimension to pixels. Therefore, whenever mode-split happens and corresponding dimensions are identified, and human interference to either recoding setup or training hyperparameters can be applied.

We can envision at least two possible future directions for Selfee. One is to optimize our designs of self-supervised learning method. On the one hand, advanced self-supervised learning methods like DINO (Caron et al., 2021) (with visual transformers, ViTs) could separate objects from the background and extract more explainable representations. Besides, by using ViTs, the neural network could be more robust against distractive textures (Naseer et al., 2021). At the same time, more temporal information can also be incorporated for a better understanding of motions. Combining these two, equipping ViTs with spatial-temporal attention could extract better features. On the other hand, although Siamese networks are popular choices for self-supervised learning, they require two times more computational resources than single-branch designs. A recent work on instance learning shed light on self-supervised learning with smaller datasets and simpler architectures. This could be a promising direction in which self-supervised learning for animal behaviors could be more accessible for biologists. In summary, possible improvements of Selfee designs can either bring in more advanced and complex architectures for better performance or try more simplified instance learning techniques to achieve easier deployment.

Another direction will be explainable behavior forecasting for a deeper understanding of animal behaviors. For a long time, behavior forecasting has been a field with extensive investigations in which RNNs, LSTMs, or transformers are usually applied (Aksan et al., 2020; Fragkiadaki, 2015; Sun et al., 2021). However, most of these works use coordinates of key points as inputs. Therefore, the trained model might predominantly focus on spatial movement information and discover fewer behavioral syntaxes. By representation learning, spatial information is essentially condensed so that more syntaxes might be highlighted. Transformer models for forecasting could capture correlations between sub-series as well as long-term trends like seasonality (Wu et al., 2021). These deep learning methods would provide behavioral neuroscientists with powerful tools to identify behavior motifs and syntaxes that organize stereotyped motifs beyond the Markov property.

## Materials and methods

**Key resources table:** 

Reagent type (species) or resource	Designation	Source or reference	Identifiers	Additional information
Genetic reagent (*Drosophila melanogaster*)	*w^1118^*	–	–	Female, Figure 3A–C & Figure 5F–G; male, Figure 4G–J
Genetic reagent (*Drosophila melanogaster*)	*CS*	–	–	Male, Figure 3A–C, Figure 4B & Figure 5F–G
Genetic reagent (*Drosophila. melanogaster*)	*CCHa1^attP^*	BDRC	84458	w[*]; TI{RFP[3xP3.cUa]=TI}CCHa1[attP]; male, Figure 4B
Genetic reagent (*Drosophila melanogaster*)	*CCHa1-R^attP^*	BDRC	84459	w[*]; TI{RFP[3xP3.cUa]=TI}CCHa1-R[attP]; male, Figure 4B
Genetic reagent (*Drosophila melanogaster*)	*CCHa2^attP^*	BDRC	84460	w[*]; TI{RFP[3xP3.cUa]=TI}CCHa2[attP]; male, Figure 4B
Genetic reagent (*Drosophila melanogaster*)	*CCHa2-R^attP^*	BDRC	84461	w[*]; TI{RFP[3xP3.cUa]=TI}CCHa2-R[attP]; male, Figure 4B
Genetic reagent (*Drosophila melanogaster*)	*CCHa2-R-RA^Gal4^*	BDRC	84603	TI{2 A-GAL4}CCHa2-R[2 A-A.GAL4]; with Kir2.1, Figure 4B
Genetic reagent (*Drosophila melanogaster*)	*CCHa2-R-RB^Gal4^*	BDRC	84604	TI{2 A-GAL4}CCHa2-R[2A-B.GAL4]; with Kir2.1, Figure 4B
Genetic reagent (*Drosophila melanogaster*)	*CNMa^attP^*	BDRC	84485	w[*]; TI{RFP[3xP3.cUa]=TI}CNMa[attP]; male, Figure 4B
Genetic reagent (*Drosophila melanogaster*)	*Oamb^attP^*	BDRC	84555	w[*]; TI{RFP[3xP3.cUa]=TI}Oamb[attP]; male, Figure 4B
Genetic reagent (*Drosophila melanogaster*)	*Dop2R^KO^*	BDRC	84720	TI{TI}Dop2R[KO]; male, Figure 4B
Genetic reagent (*Drosophila melanogaster*)	*DopEcR^Gal4^*	BDRC	84717	TI{GAL4}DopEcR[KOGal4.w-]; male, Figure 4B
Genetic reagent (*Drosophila melanogaster*)	*SerT^attP^*	BDRC	84572	w[*]; TI{RFP[3xP3.cUa]=TI}SerT[attP]; male, Figure 4B
Genetic reagent (*Drosophila melanogaster*)	*Trh^Gal4^*	BDRC	86146	w[*]; TI{RFP[3xP3.cUa]=2 A-GAL4}Trh[GKO]; male, Figure 4B
Genetic reagent (*Drosophila melanogaster*)	*TK^attP^*	BDRC	84579	w[*]; TI{RFP[3xP3.cUa]=TI}Tk[attP]; male, Figure 4B
Genetic reagent (*Drosophila melanogaster*)	*UAS-Kir2.1*	BDRC	6595	w[*]; P{w[+mC]=UAS-Hsap\KCNJ2.EGFP}7; with Gal4, Figure 4B
Genetic reagent (*Drosophila melanogaster*)	*NorpA^36^*	BDRC	9048	w[*] norpA[P24]; male, Figure 5F–G
Genetic reagent (*Drosophila melanogaster*)	*Tdc2^RO54^*	Pan Lab at SEU		Tdc2[RO54]; male, Figure 4B
Genetic reagent (*Drosophila melanogaster*)	Taotie-Gal4	Zhu Lab at IBP		w[*]; P{w[+mC]=Gr28 b.b-GAL4.4.7}10; with Kir2.1, Figure 4B
Genetic reagent (*Mus musculus*)	C57BL/6J	–	–	Figure 3D–F & Figure 5B–D
Software, algorithm	python	Anaconda	–	3.8.8
Software, algorithm	numpy	Anaconda	–	1.19.2
Software, algorithm	matplotlib	Anaconda	–	3.4.1
Software, algorithm	av	conda-forge	–	8.0.3
Software, algorithm	scipy	Anaconda	–	1.6.2
Software, algorithm	cudatoolkit	conda-forge	–	11.1.1
Software, algorithm	pytorch	pytorch	–	1.8.1
Software, algorithm	torchvision	pytorch	–	0.9.1
Software, algorithm	pillow	Anaconda	–	8.2.0
Software, algorithm	scikit-learn	Anaconda	–	0.24.2
Software, algorithm	pandas	Anaconda	–	1.2.4
Software, algorithm	lightgbm	conda-forge	–	3.2.1
Software, algorithm	opencv-python	PyPI	–	4.5.3.56
Software, algorithm	psutil	PyPI	–	5.8.0
Software, algorithm	pytorch-metric-learning	PyPI	–	0.9.99
Software, algorithm	pyhsmm	PyPI	–	0.1.6
Software, algorithm	autoregressive	PyPI	–	0.1.2
Software, algorithm	dtw-python	PyPI	–	1.1.10
Software, algorithm	SLEAP	conda-forge	–	1.2.2
Software, algorithm	DEEPLABCUT	PyPI	–	2.2.0.2

### Fly stocks

All fly strains were maintained under a 12 hr/12 hr light/dark cycle at 25°C and 60% humidity (PERCIVAL incubator). The following fly lines were acquired from Bloomington *Drosophila* Stock Center: *CCHa1^attP^* (84458), *CCHa1-R^attP^* (84459), *CCHa2^attP^* (84460), *CCHa2-R^attP^* (84461), CCHa2-R-RA^Gal4^ (84603), CCHa2-R-RB^Gal4^ (84604), *CNMa^attP^* (84485), *Oamb^attP^* (84555), *Dop2R^KO^* (84720), *DopEcR^Gal4^* (84717), *SerT^attP^* (84572), *Trh^Gal4^* (86146), *TK^attP^* (84579), *NorpA^36^* (9048), UAS-Kir2.1 (6595). *Tdc2^RO54^* was a gift from Dr Yufeng Pan at Southeast University, China. Taotie-Gal4 was a gift from Dr Yan Zhu at Institute of Biophysics, Chinese Academy of Sciences, China.

### Fly courtship behavior and male-male interaction

Virgin female flies were raised for 4–6 days in 15-fly groups, and naïve male flies were kept in isolated vials for 8–12 days. All behavioral experiments were done under 25°C and 45–50% humidity. Flies were transferred into a customized chamber of 3 mm in height and 10 mm in diameter by a homemade aspirator. Fly behaviors were recorded using a stereoscopic microscope mounted with a CCD camera (Basler ORBIS OY-A622f-DC) at the resolution of 1000×500 (for two chambers at the same time), or 640×480 (for individual chambers) and a frame rate of 30 Hz. Five types of behaviors were annotated manually, including ‘chasing’ (a male fly follows a female fly), ‘wing extension’ a male fly extends unilateral wing and orientates to the female to sing courtship son, ‘copulation attempt’ (a male fly bends its abdomen toward the genitalia of the female or the unstable state that male fly mounts on a female with its wings open), and ‘copulation’ (male fly mounts on a female in a stable posture for several minutes).

### Fly defensive behavior assay

The kicking behavior was tested based on previously reported paradigms (Li et al., 2016; Liu et al., 2020). Briefly, flies were raised in groups for 3–5 days. Flies were anesthetized on ice, and then male flies were decapitated and transferred to 35 mm Petri dishes with damped filter paper on the bottom to keep the moisture. Flies were allowed to recover for around 30 min in the dishes. The probe for stimulation was homemade from a heat-melt yellow pipette tip, and the probe’s tip was 0.3 mm. Each side of flies’ wing margin was gently touched five times, and the kicking behavior was recorded manually. The statistical analysis was performed with the Mann-Whitney test with GraphPad Prism Software.

### Social behavior assay for flies

The social distance was tested based on the previously reported method (McNeil et al., 2015). Briefly, flies were raised in groups for 3 days. Flies were anesthetized paralyzed on ice, and male flies were picked and transferred to new vials (around 40 flies per vial). Flies were allowed to recover for 1 day. The vertical triangular chambers were cleaned with 75% ethanol and dried with paper towels. After assembly, flies were transferred into the chambers by a homemade aspirator. The photos were taken after 20 min, and the positions of each fly were manually marked in ImageJ. The social distances were measured with the lateral sides of the chambers (16.72 cm) as references, and the median values of the nearest, median, and average distance of each replication are calculated. The statistical analysis was performed with the Mann-Whitney test in GraphPad Prism Software.

### Mice mating behavior assay

Wild-type mice of C57BL/6J were purchased from Slac Laboratory Animal (Shanghai). Adult (8–24 weeks of age) male mice were used for sexual behavior analysis. All animals were housed under a reversed 12 hr/12 hr light-dark cycle with water and food ad libitum in the animal facility at the Institute of Neuroscience, Shanghai, China. All experiments were approved by the Animal Care and Use Committee of the Institute of Neuroscience, Chinese Academy of Sciences, Shanghai, China (IACUC No. NA-016-2016).

Male mice were singly housed for at least 3 days prior to sexual behavioral tests. All tests were initiated at least 1 hr after lights were switched off. Behavioral assays were recorded using infrared cameras at the frame rate of 30 Hz. Female mice were surgically ovariectomized and supplemented with hormones to induce receptivity. Hormones were suspended in sterile sunflower seed oil (Sigma-Aldrich, S5007) and injected 10 mg (in 50 mL oil) and 5 mg (in 50 mL oil) of 17b-estradiol benzoate (Sigma-Aldrich, E8875) 48 and 24 hr preceding the test, respectively. On the day of the test, 50 mg of progesterone (Sigma-Aldrich, P0130; in 50 mL oil) was injected 4–6 hr prior to the test. Male animals were adapted 10 min to behavioral testing rooms where a recording chamber equipped with video acquisition systems was located. A hormonal primed ovariectomized C57BL/6J female (OVX) was introduced to the home cage of male mice and videotaped for 30 min. Mating behavior tests were repeated three times with different OVX at least 3 days apart. Videos were manually scored using a custom-written MATLAB program. The following criteria were used for behavioral annotation: active nose contacts initiated by male mouse toward the female’s genitals, body area, faces were defined collectively as ‘social interest’; male mouse climbs the back of the female and moves the pelvis were defined as ‘mount’; rhythmic pelvic movements after mount were defined as ‘intromission’; a body rigidity posture after final deep thrust were defined as ‘ejaculation’.

### Mice OFT

All experiments were performed using the principles outlined in *the Guide for the Care and Use of Laboratory Animals of Tsinghua University*. C57BL/6J male mice, aged 8–12 weeks, were used for behavior test. Mice were housed five per cage with free access to food and water and under a 12 hr light-dark cycle (light on from 7 p.m. to 7 a.m.). All mice were purchased and maintained under standard conditions by the Animal Research Centre of Tsinghua University.

All studies and experimental protocols were approved by Institutional Animal Care and Use Committee (IACUC) at Tsinghua University (No. 19-ZY1). Specifically, the OFT was conducted in an open plastic arena (50 cm × 50 cm × 40 cm). Mice were first placed in the peripheral area with their head toward the wall. Exploration time during 10 min in the peripheral and central regions, respectively, were measured using an automated animal tracking program.

The animal tracking program was coded in Python 3 with Open-CV. Each frame was first preprocessed, and then a median filter with a kernel size of 5 and a threshold of 150 was performed sequentially. Connected components with the maximum area were extracted, and the center of gravities and borders were visualized with original images for manual proofreading. Tracking results were saved in plain text format.

### Data preprocessing, augmentation, and sampling

Fly behavior videos were decomposed into frames by FFmpeg, and only the first 10,000 frames of each video were preserved and resized into images with a resolution of 224×224. For model training of *Drosophila* courtship behavior, each video was manually checked to ensure successful copulations within 10,000 frames.

Mice behavior videos were decomposed into frames by FFmpeg, and only frames of the first 30 min of each video were preserved. Frames were then preprocessed with OpenCV (Bradski, 2000) in Python. Behavior chambers in each video were manually marked, segmented, and resized into images of a resolution of 256 × 192 (mating behavior) or 500 × 500 (OFT). For background removal, the average frame of each video was subtracted from each frame, and noises were removed by a threshold of 25 and the median filter with a kernel size of 5. Finally, the contrast was adjusted with histogram equalization.

For data augmentations, crop, rotation, flip, Turbo, and color jitter were applied. For a given frame, it formed a live-frame with its preceding and succeeding frames. For flies’ behavior video, three frames were successive, and for mice, the preceding or succeeding frame is one frame away from the current frame due to their slower dynamics (Wiltschko et al., 2015). Each live-frame was randomly cropped into a smaller version containing more than 49% (70%×70%) of the original image; then the image was randomly (clockwise or anticlockwise) rotated for an angle smaller than the acute angle formed by the diagonal line and the vertical line, then the image would be vertically flipped, horizontally flipped, and/or applied the Turbo lookup table (Mikhailov, 2019) at the probability of 50%, respectively; and finally, the brightness, contrast, saturation, and hue were randomly adjusted within 10% variation. Notably, since the Turbo transformation is designed for grayscale images, for a motion-colored RGB image, each channel was transformed individually. After Turbo transformation, their corresponded channels were composited to form a new image.

For fly data sampling, all images of all videos were randomly ranked, and each batch contained 256 images from different videos. For mice data sampling, all images of each video were randomly ranked, and each batch contained 256 images from the same video. This strategy was designed to eliminate the inconsistency of recording conditions of mice that was more severe than flies.

### Selfee neural network and its training

All training and inference were accomplished on a workstation with 128 GB RAM, AMD Ryzen 7 5800×, and one NVIDIA GeForce RTX 3090. Selfee neural network was constructed based on publications and source codes of BYOL (Grill et al., 2020), SimSiam (Chen et al., 2020), and CLD (Wang et al., 2021) with PyTorch (Paszke et al., 2019). In brief, the last layer of ResNet-50 was removed, and a three-layer 2048-dimension MLP was added as the projector. Hidden layers of the projector were followed by batch normalization (BN) and ReLU activation, and the output layer only had BN. The predictor was constructed with a two-layer bottleneck MLP with a 512-dimension hidden layer and a 2048-dimension output layer. The hidden layer but not the output layer of the predictor had BN and ReLU. As for the group discriminator for CLD loss, it had only one normalized fully connected layer that projected 2048-dimension output to 1024 dimensions, followed by a customized normalization layer that was described in the paper of CLD (Wang et al., 2021). The collapse level was monitored as one minus to average standard deviation of each channel of the normalized representation multiplied by the square root of the channel number. If a collapse happens, the standard deviation becomes zero, and the collapse level should be one. If no collapse happens, each channel should obey standard normal distribution, and the average standard deviation is one. The normalization operation cancels out the square root of the channel number. In this way, the collapse level is zero.

The loss function of Selfee had two major parts. The first part was the negative cosine loss (Chen et al., 2020; Grill et al., 2020), and the second part was the CLD loss (Wang et al., 2021). For a batch of *n* samples, *Z*, *P*, *V* represented the output of projector, predictor, and group discriminator of the main branch, respectively; *Z’*, *P’*, *V’* represented the output of the reference branch; and *sg* as the stop-gradient operator. After *k*-means clustering of *V*, the centroids of *k* classes were given by *M*, and labels of each sample were provided in the one-hot form as *L*. The hyperparameter *θ* was 0.07, and *λ* was 2. The loss function was given by the following equations:

$$CosineDistance(m,n)=1-\frac{m}{{||m||}_{2}}.\frac{n}{{||n||}_{2}}$$

$$Loss_{1}=\frac{1}{2n}\sum_{i=1}^{n}CosineDistance(sg(z_{i}),p^{}\prime_{i})+\frac{1}{2n}\sum_{i=1}^{n}CosineDistance(sg(z^{}\prime_{i}),p_{i})$$

$$CrossEntropyLoss(x,l)=-x.l+log(||exp(x)|{|}_{1})$$

$$Loss_{2}=\frac{1}{2}CrossEntropyLoss\left(\frac{\nu_{i}}{\theta }.M^{}\prime^{T},l_{i}\right)+\frac{1}{2}CrossEntropyLoss\left(\frac{\nu^{}\prime_{i}}{\theta }.M^{T},l^{}\prime_{i}\right)$$

$$Loss=\;Loss_{1}\;+\;\lambda \;Loss_{2}$$

For all training processes, the Selfee network was trained for 20,000 steps with the SDG optimizer with a momentum of 0.9 and a weight decay of 1e-4. The learning rate was adjusted in the one-cycle learning rate policy (Smith and Topin, 2017) with base learning rates and a pct start of 0.025. The model for *Drosophila* courtship behavior was initialized with ResNet-50 pre-trained on the ImageNet, and the base learning rate was 0.025 per batch size of 256. As for the mating behaviors of mice, the model was initialized with weights trained on the fly dataset, and the base learning rate was 0.05 per batch size of 256.

For fly courtship behavior, 516 and 7 videos (4,607,274 and 55,708 frames) were used as train set and test set, respectively; for mice mating behavior, 118 and 13 videos (4,943,101 and 548,993 frames) were used as train set and test set, respectively. For comparison with animal tracking methods, Selfee was fine-tuned with 11 and 1 videos (1,188,550 and 108,050 frames).

### t-SNE visualization

Video frames for t-SNE visualization were all processed by Selfee. Embeddings of three tandem frames were averaged to eliminate potential noises. All embeddings were transformed using t-SNE provided in the scikit-learn (Paszke et al., 2019) package in Python without further tuning of parameters. Results were visualized with the Matplotlib (Hunter, 2007) package in Python, and their colors were assigned based on human annotations of video frames.

### Classification

Two kinds of classification methods were implemented, including the *k*-NN classifier and the LightGBM classifier. The weighed *k*-NN classifier was constructed based on the previous reports (Chen et al., 2020; Wu et al., 2018). LightGBM classifier (Ke et al., 2017) was provided by its official package in Python. The *F*_1_ score and mAP were calculated with the scikit-learn (Paszke et al., 2019) package in Python. Scores for different type of behaviors were averaged in the ‘macro’ way, while scores for behaviors of different intensity were averaged in the ‘micro’ way.

For fly behavior classification, seven 10,000-frame videos were annotated manually. Sevenfold cross-validation was performed using embeddings generated by Selfee and the *k*-NN classifier. Inferred labels were forced to be continuous through time using inferred labels of 21 neighbor frames to determine the final result. The neighborhood length was determined by plotting the similarity between neighbor frames and the current frame and choosing the frame number where similarities dropped to the half between the peak and the bottom. Then, a video independent of the cross-validation was annotated and inferred by a *k*-NN classifier using all 70,000 samples, and the last 3000 frames were used for the raster plot.

To compare different types of features, three published labeled videos were used. Each frame of video was labelled with a wing extension score from 0 to 6 (scored from 0 to 3 by two individuals). Each video of 30 min was evenly split into two parts, and six video samples were used for sixfold cross-validation. Voting by 21 neighbors was not used. FlyTracker features and JAABA features were obtained from the original publication (https://library.ucsd.edu/dc/object/bb20197654), and distances between key points were calculated from the tracking results of FlyTracker.

For rat behavior classification, the RatSI dataset (Lorbach et al., 2018) (a kind gift from Noldus Information Technology bv) contains nine manually annotated videos. We neglected three rarest annotated behaviors: moving away, nape attacking, and pinning, and we combined approaching and following into a larger category. Therefore, we used five kinds of behaviors, including allogrooming, approaching or following, social nose contact, solitary, and others. Ninefold cross-validation was performed using embeddings generated by Selfee and the *k*-NN classifier. Inferred labels were forced to be continuous through time by using inferred labels of 81 neighbor frames to determine the final result.

For mice behavior classification, eight videos were annotated manually. Eightfold cross-validation was performed using embeddings generated by Selfee and the *k*-NN classifier. To incorporate more temporal information, the LightGBM classifier and additional features were also used. Additional features include slide moving average and standard deviation of 81-frame time windows, the main frequencies, and their energy (using short-time Fourier transform in SciPy; Virtanen et al., 2020) within 81-frame time windows. Early-stop was used to prevent over-fitting. Inferred labels were forced to be continuous through time by using inferred labels of 81 neighbor frames to determine the final result. Then, a video independent of the cross-validation was annotated and inferred by an ensemble classifier of eight previously constructed classifiers, and all frames were used for the raster plot.

### Anomaly detection

For a group of query embeddings of sequential frames *q*_1_, *q*_2_, *q*_3_, …, *q_n_*, and a group of reference embeddings of sequential frames *r*_1_, *r*_2_, *r*_3_, …, *r_m_*, the anomaly score of each query frame was given by the following equation:

$$AnomalyScore(q_{i})={min}_{j=1}^{m}\left(CosineDistance(q_{i},r_{j})\right)-{min}_{|j-i|}<50\left(CosineDistance(q_{i},q_{j})\right)$$

A PyTorch implementation of cosine similarity (Musgrave et al., 2020) was used for accelerated calculations.

The anomaly score of each video was the average anomaly score of the top 100 anomalous frames. The statistical analysis of the genetic screening was performed with one-way ANOVA with Benjamini and Hochberg correction in GraphPad Prism Software.

If negative controls are provided, anomalous frames are defined as frames with higher anomaly scores than the maximum anomaly score of frames in negative control videos.

### Autoregressive hidden Markov model

All AR-HMMs were built with the implementation of MoSeq (Wiltschko et al., 2015) (https://github.com/mattjj/pyhsmm-autoregressive; Linderman, 2018). A PCA model that could explain 95% of variance of the control group was built and used to transform both control and experiment groups. The max module number was set as 10 for all experiments unless indicated otherwise. Each model was sampled for 1000 iterations. We kept other hyperparameters the same as the examples provided by this package. State usages of each module in control and experimental groups were analyzed by Mann-Whitney test with SciPy Virtanen et al., 2020 followed with Benjamini and Hochberg correction. The state usages were also visualized after PCA dimensional reduction with scikit-learn (Paszke et al., 2019) and Matplotlib (Hunter, 2007) for the exclusion of possible obvious outliners or batch effects.

### Dynamic time warping

DTW was modified from the Python implementation (Toni, 2009) (https://dynamictimewarping.github.io/python/). Specifically, PyTorch implementation of cosine similarity (Musgrave et al., 2020) was used for accelerated calculations.

### Pose estimation with SLEAP

We used the official implementation of SLEAP. For explanation for t-SNE plot of Selfee features, we labeled the same 3000 frames with the help of SLEAP. First, we labeled around 400 frames and trained a SLEAP model. The trained SLEAP model was used to infer all 3000 frames, tracking was performed using the ‘simple’ method, and the target number of instances per frame was set to 2. Inferred skeletons and tracking results were manually proofread and corrected. These results were used for the following analysis of the t-SNE plot. Then, all 3000 frames were used to train a new SLEAP model. This new model was used for the pose estimation of another clip of a fly courtship video (shown in Figure 1—videos 1–3 and Figure 1—figure supplement 3).

### Visualization tracking results from FlyTracker

Tracking results of the Fly-vs-Fly dataset were obtained from its official website. The head and tail coordinates were calculated from the center, the orientation, and the major axis length. Tracking results were visualized with OpenCV.

### Pose estimation with DeepLabCut

We used the official implementation of DeepLabCut (Lauer et al., 2021; Mathis et al., 2018). For training, 120 frames of a mating behavior video were labeled manually, and 85% were used as the training set. Marked body parts included nose, ears, body center, hips, and bottom, following previous publications (Segalin et al., 2020; Sun et al., 2021). The model (ResNet-50 as the backbone) was trained for 100,000 iterations, with a batch size of 16. We kept other hyperparameters the same as default settings.

### Data and code availability statement

Major datasets that support the findings of this study are available from Dryad (https://doi.org/10.5061/dryad.brv15dvb8). Other data are available from the corresponding author upon reasonable request, because they are too large to upload to a particular server. Source codes used in this article are available on GitHub (https://github.com/EBGU/Selfee; Jia, 2022; copy archived at swh:1:rev:3af2d1ed2dfcf3bd1d1c18d488ed87f7b826529c).

## Data Availability

Major data used in this study were uploaded to Dryad, including pretrained weights. Data can be accessed via: https://doi.org/10.5061/dryad.brv15dvb8. With the uploaded dataset and pretrained weights, our experiments can be replicated. However, due to its huge size and the limited internet service resources, we are currently not able to share our full training dataset. The full dataset is as large as 400GB, which is hard to upload to a public server and will be difficult for others users to download. The training dataset, is available from the corresponding author upon reasonable request (https://www.ie.tsinghua.edu.cn/eng/info/1017/1347.htm), and then we can discuss how to transfer the dataset. No project proposal is needed as long as the dataset is not used for any commercial purpose. Our Python scripts can be accessed on GitHub: https://github.com/EBGU/Selfee, copy archived at swh:1:rev:3af2d1ed2dfcf3bd1d1c18d488ed87f7b826529c. Other software used in our project include ImageJ (https://imagej.net/software/fiji/) and GraphPad Prism (https://www.graphpad.com/). All data used to plot graphs and charts in the manuscript can be fully accessed on Dryad (DOI 10.5061/dryad.brv15dvb8). The following dataset was generated: JiaY LiS GuoX LeiB HuJ XuX ZhangW 2022Data from: Selfee: Self-supervised features extraction of animal behaviorsDryad Digital Repository10.5061/dryad.brv15dvb8PMC929613235708244 The following previously published datasets were used: EyrunE BransonS Burgos-ArtizzuP HoopferED SchorJ AndersonDJ PeronaP 2021Fly-vs-FlyCaltechDATA1893 XuboL MargotW KenichiI PavanN KentaA 2020Data from: Quantifying influence of human choice on the automated detection of *Drosophila* behavior by a supervised machine learning algorithmLIBRARY DIGITAL COLLECTIONS10.6075/J0QF8RDZPMC774394033326445 LorbachM KyriakouEI PoppeR van DamEA NoldusLPJJ VeltkampRC 2017RatSINoldus Information Technologyratsi-dataset

## Additional files

MDAR checklist
